# Post-marketing safety concerns with dolutegravir: a pharmacovigilance study based on the FDA adverse event reporting system database

**DOI:** 10.3389/fphar.2025.1625601

**Published:** 2025-07-30

**Authors:** Juan Su, Long He, Menglei Wang

**Affiliations:** ^1^ Pharmacy Department, The Second People’s Hospital of Meishan City, Meishan, China; ^2^ School of Pharmacy, Southwest Medical University, Luzhou, China; ^3^ Medical Affairs Department, The Second People’s Hospital of Meishan City, Meishan, China

**Keywords:** dolutegravir, FAERS, adverse events, pharmacovigilance, HIV

## Abstract

**Background and aims:**

Antiretroviral therapy (ART) based on dolutegravir (DTG) has emerged as a critical component in the treatment of HIV infection and is widely utilized in clinical practice. However, the existing post-marketing pharmacovigilance studies on DTG are incomplete; therefore, this study aims to comprehensively analyze the adverse events (AEs) related to DTG by utilizing the FDA Adverse Event Reporting System (FAERS) database.

**Methods:**

This study includes adverse event reports from the fourth quarter of 2013 to the fourth quarter of 2024 in the FAERS database. Four disproportionality analysis methods were employed for adverse event signal mining: Reporting Odds Ratio (ROR), Proportional Reporting Ratio (PRR), Bayesian Confidence Propagation Neural Network (BCPNN), and Empirical Bayesian Geometric Mean (EBGM). Additionally, clinical priority and time-to-onset characteristics were assessed.

**Results:**

A total of 13,007 case reports were collected with DTG as the primary suspected (PS) drug, including 32,383 AEs. Observed disproportionate associations with the use of DTG were related to pregnancy, puerperium and perinatal conditions (*n* = 688; ROR = 5.53, 95% CI 5.13–5.96), hepatobiliary disorders (*n* = 655; ROR = 2.45, 95% CI 2.27–2.65), and congenital, familial and genetic disorders (*n* = 550; ROR = 6.11, 95% CI 5.62–6.65). A total of 341 AE signals were identified in the overall analysis, among which 164 were rated as moderate clinical priority, with no high clinical priority signals. Notably, subgroup analyses revealed that progressive multifocal leukoencephalopathy (*n* = 9) in males, progressive multifocal leukoencephalopathy (*n* = 6) and deafness neurosensory (*n* = 3) in the 18–45 age group, and hepatic necrosis (*n* = 4) in the 46–65 age group were rated as high clinical priority. The overall onset time for all AEs exhibited an early failure pattern, with a median onset time of 74 days (IQR 19–310.5), whereas the median onset time for designated medical events (DMEs) was 59 days (IQR 12–186).

**Conclusion:**

The long-term safety of DTG requires reassessing its risk-benefit ratio. To mitigate the risk of irreversible organic damage, a structured risk management strategy is essential. This strategy should encompass restrictions on contraindicated populations, enhanced monitoring of high-risk subgroups, and the implementation of post-marketing prospective cohort studies to ensure the sustainability of antiviral therapy.

## 1 Introduction

The global public health issue posed by HIV infection and transmission remains critical. According to statistics, approximately 1.3 million individuals were newly infected with HIV globally in 2023, and around 630,000 succumbed to AIDS-related illnesses (Global HIV & AIDS statistics—Fact sheet UNAIDS, 2025). Advances in antiretroviral therapy have transformed HIV and opportunistic infections into manageable chronic conditions. By the end of 2023, approximately 77% (30.7 million) of people living with HIV (PLWH) were receiving antiretroviral treatment (Swinkels et al., 2024; Data on the HIV response, 2025). Dolutegravir (DTG), a second-generation HIV-1 integrase strand transfer inhibitor (INSTI), is distinguished by its potent efficacy, high resistance barrier, once-daily pharmacokinetics, low potential for drug interactions, and reliable safety profile (Cahn et al., 2013; Raffi et al., 2013; Walmsley et al., 2013; Castagna et al., 2014; Mehta et al., 2023; Cordova et al., 2025; White et al., 2025). It has been recommended by international treatment guidelines as a first-line therapy for both antiretroviral therapy (ART)-naive and treatment-experienced patients since 2014 (Gandhi et al., 2023; What’s New, 2024; EACS Guidelines 2024, n.d.).

The Botswana Tsepamo study previously reported that among 426 infants born to mothers who received DTG treatment prior to pregnancy, 4 (0.94%) were diagnosed with neural tube defects (NTD), a rate that is higher than expected (Zash et al., 2018). Subsequent pharmacovigilance studies conducted detailed analyses but did not establish a definitive link between DTG and the occurrence of neural tube defects (van De Ven et al., 2020; Saint-Lary et al., 2024). Meanwhile, as the number of pre-pregnancy exposures increased, the risk of NTD significantly decreased by 17% (Zash et al., 2019), and other studies collectively enhanced the credibility of these findings (Eke et al., 2023; Gill et al., 2023; Kourtis et al., 2023). The potential adverse effects of DTG continue to emerge. As safety concerns regarding neural tube defects gradually diminish, issues related to weight gain and cardiovascular risks have come to the forefront (Brennan et al., 2023; Palella et al., 2023). However, the evaluation of DTG’s effects on weight changes and cardiovascular risks is often complicated by various influencing factors, including the impact of HIV infection on body metabolism and the alterations in clinical ART regimens (Ebasone et al., 2025). Recent research suggests that weight gain following the use of INSTIs is more likely associated with changes in ART regimens rather than the medication itself (Wu et al., 2024; Cutshaw et al., 2025).

Neuropsychiatric adverse events associated with DTG are of considerable significance. A systematic review by Pérez-Valero et al. examined discontinuation rates due to neuropsychiatric symptoms in HIV-infected individuals receiving INSTIs. The findings indicated that the discontinuation rate among patients on DTG-based regimens ranged from 0% to 11.8% (median 2.8%), whereas those receiving Bictegravir/Emtricitabine/Tenofovir Alafenamide (BIC/FTC/TAF) exhibited a discontinuation rate ranging from 0% to 3.1% (median 0.7%), highlighting a significant difference. Notably, factors such as age over 50 years, female sex, and lack of prior ART were identified as potential risk factors (Pérez-Valero et al., 2023). The use of DTG has frequently been linked to adverse events, including depression and insomnia (Hoffmann and Llibre, 2019; Pérez-Valero et al., 2023; Chanie et al., 2025), with female HIV patients being particularly vulnerable to neuropsychiatric complications, particularly depression, which has increasingly attracted research attention. In a study evaluating depression and suicidal behavior associated with integrase inhibitors (INTIs) conducted by Laure-Hélène Préta et al., notable disparities in information were highlighted, especially regarding DTG, which exhibited a higher risk of depression (Reporting Odds Ratio [ROR] 1.3; 95% Confidence Interval [CI]: 1.1–1.6) and suicide (ROR 1.8; 95% CI: 1.5–2.3) compared to other ART (Préta et al., 2023).

The FDA Adverse Event Reporting System (FAERS) database serves as an open-access post-marketing safety surveillance resource, providing essential information for pharmacovigilance research. Our study aims to leverage the extensive real-world data accumulated post-marketing of DTG to enhance the safety monitoring of DTG while thoroughly investigating potential adverse event signals, thereby offering valuable references for the individualized selection and adjustment of clinical ART regimens.

## 2 Materials and methods

### 2.1 Data sources

**FIGURE 1:**
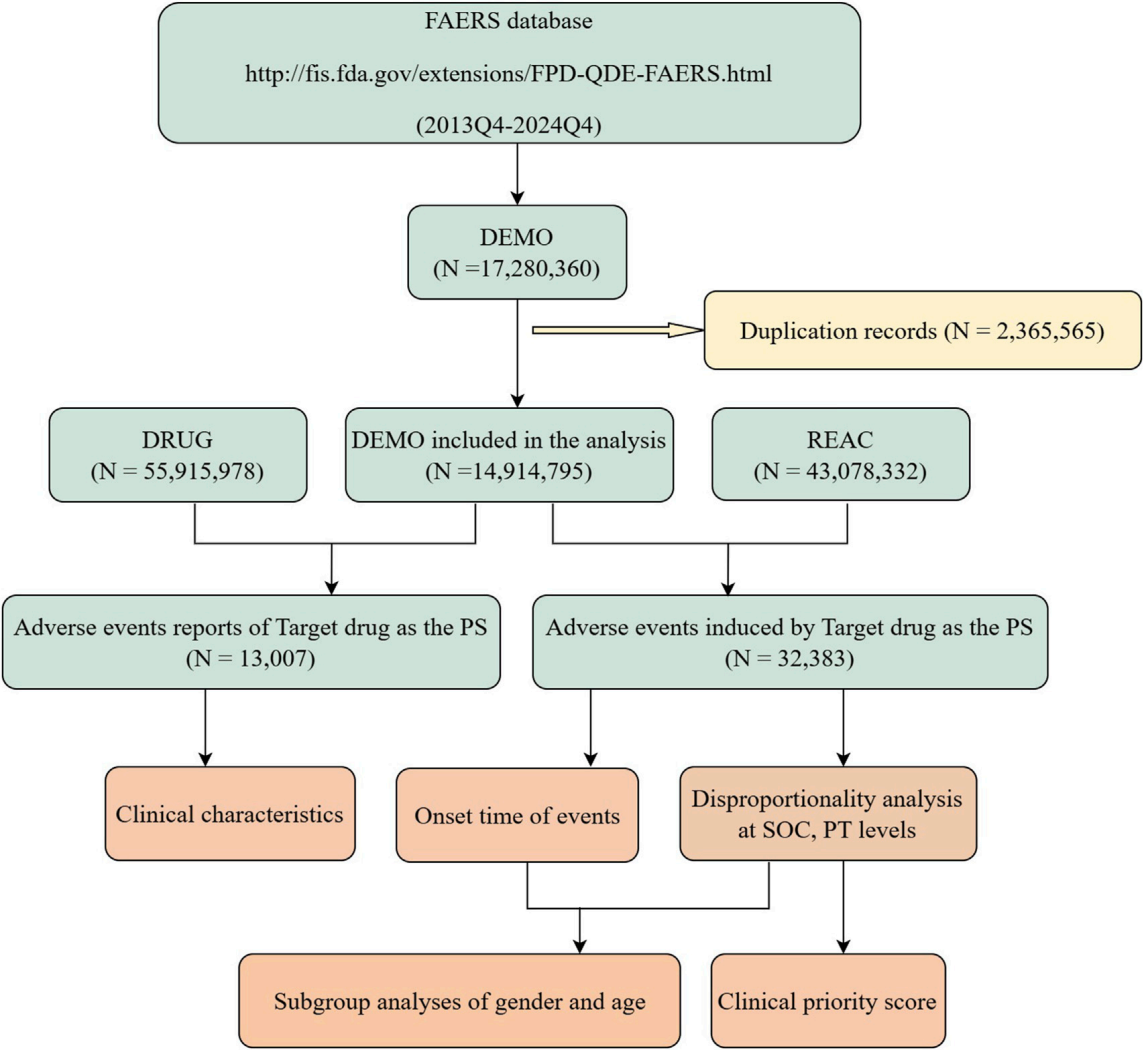
Flow chart of data extraction and analysis. A detailed description of the flow chart for data extraction and analysis of adverse events for dolutegravir in the US Food and Drug Administration Adverse Event Reporting System (FAERS).

The data extraction and analysis procedure is illustrated in Figure 1. Data were extracted from the FAERS (https://fis.fda.gov/extensions/FPD-QDE-FAERS/FPD-QDE-FAERS.html), which primarily includes reports of adverse events, medication errors, and product quality complaints submitted by healthcare professionals, pharmaceutical manufacturers, legal representatives, and individual patients. This dataset encompasses patient demographic data (DEMO), drug information (DRUG), adverse event information (REAC), patient outcome information (OUTC), report source information (RPSR), drug therapy date information (THER), and drug indication (INDI). Following the removal of duplicate reports using the recommended methodology (He et al., 2025), we screened adverse event reports from the fourth quarter of 2013 to the fourth quarter of 2024, including only those reports where DTG was the primary suspected (PS) drug. This approach enhances the accuracy of the analysis and minimizes potential confounding factors.

### 2.2 Disproportionate analysis

Disproportionality analysis is a widely utilized method for monitoring adverse drug reaction signals in the post-marketing phase. In this study, we employed four disproportionality analysis methods to identify AE signals associated with drugs: the Reporting Odds Ratio (ROR), Proportional Reporting Ratio (PRR), Empirical Bayesian Geometric Mean (EBGM), and Bayesian Confidence Propagation Neural Network (BCPNN). Detailed formulas and signal detection criteria are provided in the Supplementary Table S1; only those signals that met the detection thresholds of all four methods were considered potential AE signals for DTG. Adverse events were standardized according to the Medical Dictionary for Regulatory Activities (MedDRA v27.1), and signal mining was conducted at both the System Organ Class (SOC) and Preferred Term (PT) levels. Furthermore, to explore potential differences in AE signals among various populations following the use of DTG and to enhance the robustness of our results, we also performed subgroup analyses based on age and gender.

### 2.3 Classification and prioritization of relevant disproportionality signals

To evaluate the clinical priority of positive signals, we assessed four indicators: the number of target events, the lower limit of the ROR, the proportion of reports with death as outcome, and compliance with the Important Medical Event (IME) or Designated Medical Event (DME) criteria (Cecco et al., 2024) (see Table 1).

**TABLE 1:** Criteria and relevant scores to prioritize AEs emerged from disproportionality analysis.

Criterium	2 points	1 point	0 point
Reporting rate (cases/non-cases)	>10%	1%–10%	0%–1%
Signal stability (consistency across disproportionality analyses)	3 of 3	2 of 3	1 of 3
Fatal report proportion (proportion of reports with death as outcome)	>50%	25%–50%	<25%
Clinical relevance (serious likely drug-attributable AEs)	DME	IME	None

AEs, adverse events; DME, designated medical event; IME, important medical event.

Scores ranging from 0 to 2, 3 to 5, and 6 to 8 were used to classify AEs as having low, moderate, or high priority, respectively.

### 2.4 Analysis of onset time and statistical analysis

The time interval from the start date to the event date in the AE reports was utilized to determine the time to onset. Reports with missing information were excluded from the analysis, followed by a Kaplan-Meier analysis. Subgroup analyses were conducted separately based on age, sex, medical event category, and report outcome. This involved calculating the median time to onset and the interquartile range (IQR), with differences between groups tested using the Wilcoxon test, and statistical significance was set at *p* < 0.05. This study employed R (version 4.3.3) and Microsoft Excel (version 2021) for data processing and analysis, with results visualized in GraphPad Prism (version 9.5).

## 3 Descriptive analysis

A total of 13,007 case reports associated with the use of DTG were included in this analysis, encompassing 32,383 reported adverse events (see Figure 1). The AE characteristics of DTG are summarized in Table 2. The majority of these reports originated from the United States (59.11%), with a higher proportion of reports submitted by males (52%) compared to females (21%). The number of reports submitted by individuals aged 18–45 and 46–65 was notably higher and similar (19.59% vs. 18.87%), with a median age of 47 years (IQR 35–57). Additionally, consumers submitted the majority of reports (44.90%), and among the known report outcomes, other serious events (important medical events) accounted for the highest proportion (30.00%), followed by hospitalization (13.60%). The majority of patients utilized DTG for the treatment of HIV infection.

**TABLE 2:** Reported characteristics of adverse events for dolutegravir.

Characteristics	Case Number, n	Proportions, %
Gender		
F	2,731	21.00%
M	6,766	52.00%
Missing	3,510	27.00%
Age (years)		
<18	123	0.95%
18–45	2,548	19.59%
46–65	2,455	18.87%
>65	492	3.78%
Missing	7,389	56.81%
Reporter country (top 5)		
United States	7,684	59.11%
Japan	789	6.07%
France	641	4.93%
United Kingdom	562	4.32%
Italy	388	2.98%
Others	2,943	22.63%
Occupation of reporters		
Consumer	5,844	44.90%
Physician	3,261	25.10%
Health Professional	1,788	13.70%
Pharmacist	1,372	10.50%
Other health-professional	517	4.00%
Lawyer	13	0.10%
Missing	212	1.60%
Outcomes		
Congenital anomaly	288	2.20%
Death	733	5.60%
Disability	90	0.70%
Hospitalization - Initial or Prolonged	1,766	13.60%
Life-threatening	272	2.10%
Other serious (important medical event)	3,907	30.00%
Required intervention to prevent permanent impairment/damage	20	0.20%
Missing	5,931	45.60%
Indications (top 5)		
HIV infection	7,569	58.20%
Product used for unknown indication	3,810	29.30%
HIV infection CDC group iii	118	0.90%
Antiretroviral therapy	118	0.90%
Acquired immunodeficiency syndrome	100	0.80%
Missing	835	6.40%

**FIGURE 2:**
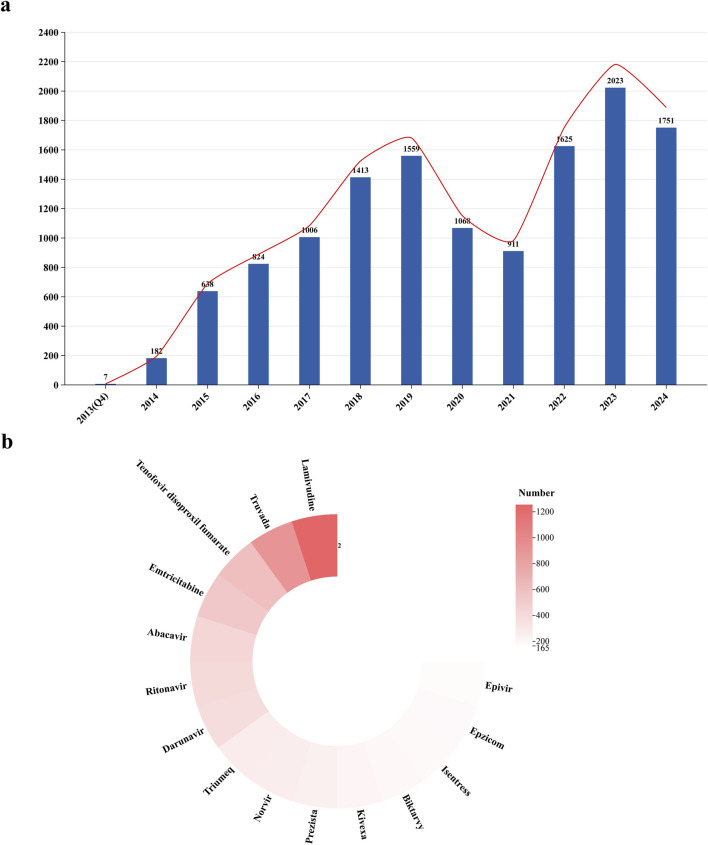
Trends in the number of AEs reported for dolutegravir from Q4 2013 to Q4 2024 and analysis of co-administration. **(a)** The report trend of dolutegravir. **(b)** Analysis of co-administration.

Figure 2a illustrates the trend in the number of reported cases from 2013 to 2024. Until 2019, the number of reports exhibited a consistent upward trajectory; however, it experienced a sharp decline post-2019, followed by a steep increase after 2021. In the statistical analysis of drug combinations with DTG (Figure 2b), Lamivudine was the most frequently co-administered drug (*n* = 1,256), followed by Truvada (*n* = 915) and Tenofovir Disoproxil Fumarate (*n* = 606).

Among the 27 SOC categories involved, the following met the criteria for signal detection and were identified as positive SOC signals: pregnancy, puerperium, and perinatal conditions (*n* = 688; ROR = 5.53, 95% CI = 5.13–5.96); hepatobiliary disorders (*n* = 655; ROR = 2.45, 95% CI = 2.27–2.65); and congenital, familial, and genetic disorders (*n* = 550; ROR = 6.11, 95% CI = 5.62–6.65) (Table 3).

**TABLE 3:** Positive signals were detected by disproportionality at the soc level.

SOC	*N*	ROR (95% Cl)	PRR (Chi-squared)	EBGM (EBGM05)	IC (IC025)
Pregnancy, puerperium and perinatal conditions	688	5.53 (5.13–5.96)	5.43 (2487.9)	5.41 (5.08)	2.44 (2.33)
Hepatobiliary disorders	655	2.45 (2.27–2.65)	2.42 (549.33)	2.42 (2.27)	1.27 (1.16)
Congenital, familial and genetic disorders	550	6.11 (5.62–6.65)	6.03 (2302.12)	6 (5.59)	2.59 (2.46)

SOC, system organ class; N, the report number; ROR, the reporting odds ratio; PRR, the proportional reporting ratio; IC, the information component; EBGM, the empirical bayesian geometric mean; CI, confidence interval; 95% CI, two-sided for ROR; IC025 and EBGM05, lower one-sided for IC, and EBGM.

**FIGURE 3:**
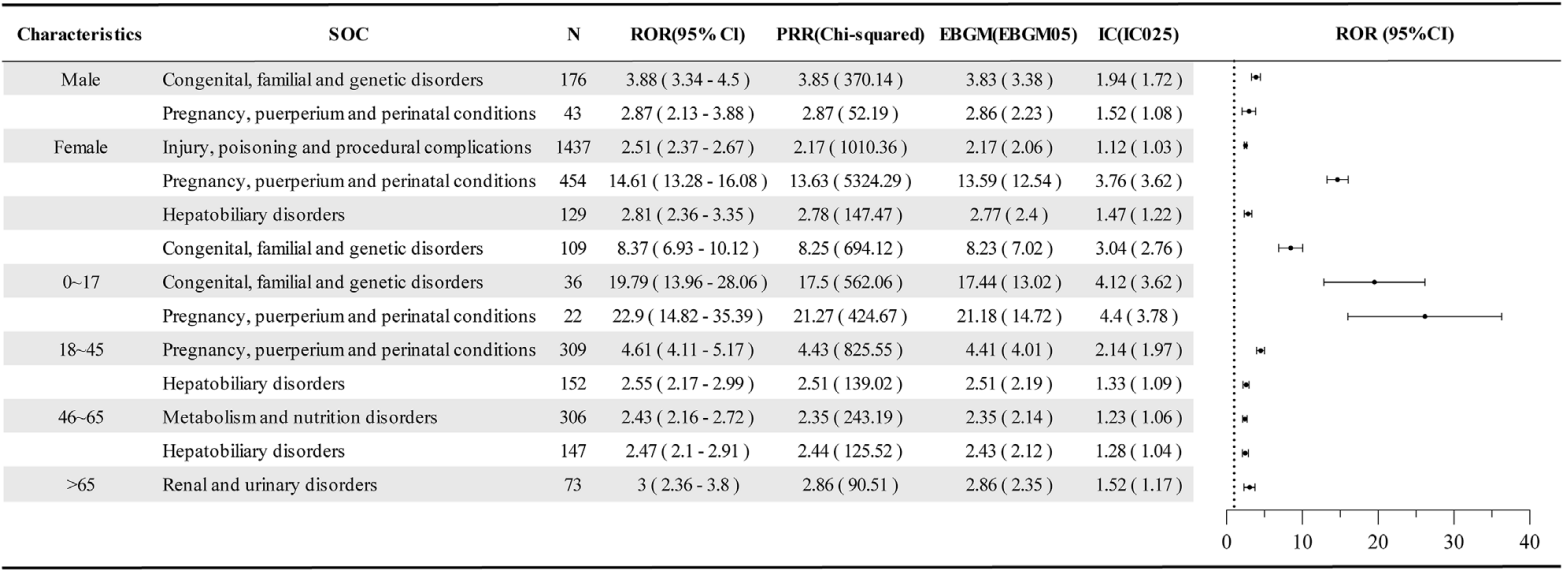
Disproportionate analysis of AE reports in the age and sex subgroups of dolutegravir. SOC, system organ class; N, the report number; ROR, the reporting odds ratio; PRR, the proportional reporting ratio; IC, the information component; EBGM, the empirical bayesian geometric mean; CI, confidence interval; 95% CI, two-sided for ROR; IC025 and EBGM05 lower one-sided for IC, and EBGM.

To enhance the robustness of signal detection results at the SOC level, further subgroup analyses including age and gender were conducted, revealing positive signals (Figure 3). Compared to the positive signal analysis results at the SOC level (Table 3), no positive signals for hepatobiliary disorders were detected in males, while a new signal for injury, poisoning, and procedural complications was identified in females. Additionally, the signal strengths for pregnancy, puerperium, and perinatal conditions (*n* = 454; ROR = 14.61, 95% CI = 13.28–16.08) and congenital, familial, and genetic disorders (*n* = 109; ROR = 8.37, 95% CI = 6.93–10.12) were significantly higher with narrower confidence intervals, suggesting that these may have been diluted in the overall assessment. In the analysis of age subgroups, a phased change was observed. In the 0–17 years group, there was a higher incidence of adverse events related to congenital, familial, and genetic disorders (*n* = 36; ROR = 19.79, 95% CI = 13.96–28.06) and pregnancy, puerperium, and perinatal conditions (*n* = 22; ROR = 22.9, 95% CI = 14.82–35.39), exhibiting high signal intensity but wide confidence intervals. The positive signals in the 18–45, 46–65, and >65 age groups gradually transitioned from congenital, familial, and genetic disorders to pregnancy, puerperium, and perinatal conditions, hepatobiliary disorders, and renal and urinary disorders.

Through disproportionality analysis, a list of AE signals was generated, these signals were subsequently matched against clinical priority criteria (Table 1) and screened, yielding 341 pharmacovigilance signals of which 164 AEs were classified as moderate clinical priority, with no high clinical priority signals identified (Supplementary Table S2). Among these, the following AEs were rated with a score of five points: progressive multifocal leukoencephalopathy (*n* = 24), hepatic necrosis (*n* = 11), anencephaly (*n* = 9), spina bifida (*n* = 9), fetal cardiac disorder (*n* = 5), gastroschisis (*n* = 4), central nervous system immune reconstitution inflammatory response (*n* = 3), and neonatal death (*n* = 3).

Additionally, there are five DME signals categorized under moderate clinical priority (Table 4), which include renal failure (4 points), drug-induced liver injury (4 points), pancreatitis (4 points), progressive multifocal leukoencephalopathy (5 points), and hepatic necrosis (5 points).

**TABLE 4:** Designated medical events showing statistically significant disproportionality in overall AEs.

PT	*N*	Signal stability				Fatal report proportion (%)	Priority level (score)
		ROR (95% Cl)	PRR (Chi-squared)	EBGM (EBGM05)	IC (IC025)		
Renal failure	174	2.58 (2.22–3)	2.57 (167.37)	2.57 (2.27)	1.36 (1.14)	15/174 (8.62%)	Moderate (4)
Drug-induced liver injury	72	4.01 (3.18–5.05)	4 (161.51)	3.99 (3.29)	2 (1.66)	7/72 (9.72%)	Moderate (4)
Pancreatitis	65	2.89 (2.26–3.68)	2.88 (79.87)	2.88 (2.35)	1.53 (1.17)	3/65 (4.62%)	Moderate (4)
Progressive multifocal leukoencephalopathy	24	5.4 (3.62–8.07)	5.4 (85.7)	5.38 (3.85)	2.43 (1.85)	10/24 (41.67%)	Moderate (5)
Hepatic necrosis	11	9 (4.97–16.29)	9 (77.69)	8.94 (5.45)	3.16 (2.32)	3/11 (27.27%)	Moderate (5)

PT, preferred term.

We replicated the same analytical method for subgroups, and Table 5 presents the DME signals detected in the subgroup analysis. In the male group, moderate clinical priority signals included pancreatitis (4 points), drug-induced liver injury (4 points), and hepatic necrosis (5 points). Notably, progressive multifocal leukoencephalopathy (6 points) exhibited a mortality rate of 55.56% and was classified as a high clinical priority. In the female group, five DMEs were identified: drug-induced liver injury (4 points), pancreatitis (4 points), acute pancreatitis (4 points), acute hepatic failure (4 points), and hepatic necrosis (4 points), all rated as moderate clinical priority. Regarding the age subgroups, no DME signals were detected in the 0–17 years group. In the 18–45 years group, two high clinical priority signals were identified: progressive multifocal leukoencephalopathy (6 points) and deafness neurosensory (6 points), alongside three moderate clinical priority signals: drug-induced liver injury (4 points), pancreatitis (4 points), and hepatic necrosis (4 points). Additionally, hepatic necrosis (6 points) was recognized as a high clinical priority signal in the 46–65 age group, along with four moderate clinical priority signals: renal failure (5 points), drug-induced liver injury (5 points), rhabdomyolysis (4 points), and generalized exfoliative dermatitis (4 points). In the >65 age group, only renal failure (5 points) and pancreatitis (4 points) were detected.

**TABLE 5:** Designated medical events showing statistically significant disproportionality in subgroups.

Characteristics	PT	*N*	Signal stability				Fatal report proportion (%)	Priority level (score)
			ROR (95% Cl)	PRR (Chi-squared)	EBGM (EBGM05)	IC (IC025)		
Male	Pancreatitis	36	2.89 (2.08–4.01)	2.88 (44.21)	2.88 (2.19)	1.53 (1.05)	3/36 (8.33%)	Moderate (4)
	Drug-induced liver injury	32	3.5 (2.47–4.95)	3.49 (56.78)	3.48 (2.61)	1.8 (1.3)	6/32 (18.75%)	Moderate (4)
	Progressive multifocal leukoencephalopathy	9	3.81 (1.98–7.34)	3.81 (18.58)	3.8 (2.2)	1.93 (1.01)	5/9 (55.56%)	High (6)
	Hepatic necrosis	6	10.17 (4.55–22.74)	10.17 (49.05)	10.07 (5.13)	3.33 (2.23)	2/6 (33.33%)	Moderate (5)
Female	Drug-induced liver injury	18	6.11 (3.84–9.71)	6.09 (76.56)	6.09 (4.13)	2.61 (1.94)	1/18 (5.56%)	Moderate (4)
	Pancreatitis	15	3.91 (2.36–6.49)	3.9 (32.4)	3.9 (2.55)	1.96 (1.24)	0	Moderate (4)
	Pancreatitis acute	7	4.5 (2.14–9.45)	4.5 (19.01)	4.49 (2.42)	2.17 (1.15)	0	Moderate (4)
	Acute hepatic failure	5	4.39 (1.83–10.56)	4.39 (13.07)	4.38 (2.1)	2.13 (0.95)	0	Moderate (4)
	Hepatic necrosis	3	12.66 (4.08–39.35)	12.66 (32.1)	12.62 (4.89)	3.66 (2.21)	0	Moderate (4)
18–45	Drug-induced liver injury	24	4.6 (3.08–6.88)	4.59 (67.07)	4.57 (3.27)	2.19 (1.61)	3/24 (12.50%)	Moderate (4)
	Pancreatitis	13	3.32 (1.92–5.72)	3.31 (20.93)	3.3 (2.09)	1.72 (0.95)	1/13 (7.69%)	Moderate (4)
	Progressive multifocal leukoencephalopathy	6	4.72 (2.12–10.53)	4.72 (17.49)	4.7 (2.4)	2.23 (1.14)	4/6 (66.67%)	High (6)
	Hepatic necrosis	6	11.77 (5.26–26.34)	11.76 (58.41)	11.64 (5.93)	3.54 (2.44)	0	Moderate (4)
	Deafness neurosensory	3	7.93 (2.54–24.69)	7.92 (18.01)	7.87 (3.04)	2.98 (1.53)	2/3 (66.67%)	High (6)
46–65	Renal failure	61	4.76 (3.7–6.13)	4.72 (178.73)	4.71 (3.81)	2.24 (1.87)	5/61 (8.20%)	Moderate (5)
	Drug-induced liver injury	14	3.67 (2.17–6.2)	3.66 (27.03)	3.66 (2.36)	1.87 (1.12)	4/14 (28.57%)	Moderate (5)
	Rhabdomyolysis	13	3.67 (2.13–6.33)	3.66 (25.12)	3.66 (2.32)	1.87 (1.1)	0	Moderate (4)
	Hepatic necrosis	4	14.07 (5.26–37.65)	14.06 (48.11)	13.95 (6.12)	3.8 (2.5)	3/4 (75.00%)	High (6)
	Dermatitis exfoliative generalised	3	7.32 (2.35–22.76)	7.32 (16.29)	7.29 (2.82)	2.87 (1.42)	0	Moderate (4)
>65	Renal failure	15	5.71 (3.43–9.51)	5.65 (57.48)	5.65 (3.68)	2.5 (1.77)	2/15 (13.33%)	Moderate (5)
	Pancreatitis	4	6.9 (2.58–18.43)	6.88 (20.1)	6.88 (3.02)	2.78 (1.49)	0	Moderate (4)

**FIGURE 4:**
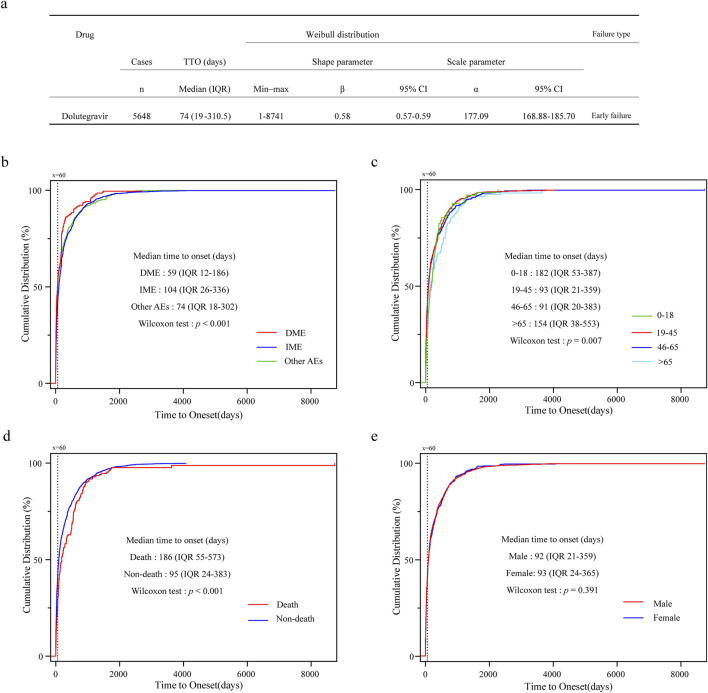
Time to onset analysis of AE for dolutegravir. **(a)** Analysis of Weibull 3-parameter for overall AEs; **(b)** Cumulative distribution curves for DME, IME, and other AEs. **(c)** Cumulative distribution curves for different age groups. **(d)** Cumulative distribution curves for fatal and non-fatal outcomes. **(e)** Cumulative distribution curves for male and female groups; IQR, interquartile range.

After excluding reports lacking information on the time of onset, a total of 5,648 cases were included and subjected to Weibull 3-parameter analysis, as well as an analysis of AE onset characteristics across different subgroups (Figure 4). The median time to onset for all included reports was 74 days (IQR 19–310.5), with the shape parameter β of the Weibull distribution and its upper limit of the 95% confidence interval both being less than 1, indicating an early failure type (Figure 4a). The cumulative distribution curves for DME, IME, and other AEs demonstrated that the median time to onset of DME was 59 days (IQR 12–186), which occurred significantly earlier than that of the other AEs (*p* < 0.001) (Figure 4b). In the analysis of the four age groups, the median onset times for the 0–18 and >65 age groups were 182 days (IQR 53–387) and 154 days (IQR 38–553), respectively, both of which were significantly delayed compared to the other two age groups (*p* = 0.007) (Figure 4c). Additionally, the median time to onset for AE reports with a fatal outcome was 186 days (IQR 55–573), while for non-fatal reports it was 95 days (IQR 24–383), indicating a significant difference (*p* < 0.001) (Figure 4d). The analysis of the reported gender subgroups did not reveal any differences in onset time between males and females (*p* = 0.39) (Figure 4e). In summary, except for the median onset time of DME being approximately 60 days, the median onset times in other subgroups were all greater than 90 days.

## 4 Discussion

This study systematically evaluates the safety profile of DTG based on data from the global spontaneous reporting system. Utilizing a substantial number of AE reports from the FAERS database, we thoroughly explored the potential AE signals and characteristics of AEs induced by DTG through disproportionality analysis and further clinical priority assessment, while also conducting an analysis of the onset time of AEs.

Our findings indicate that males reported significantly more AE reports compared to females (52% vs. 21%). According to statistics from the Joint United Nations Programme on HIV/AIDS (UNAIDS), over 73% of new HIV infections in 2023 in regions outside of sub-Saharan Africa were among males (Global HIV & AIDS statistics—Fact sheet UNAIDS, 2025). Moreover, due to concerns regarding neural tube defects in infants born to women who took DTG at conception, the World Health Organization (WHO) issued interim guidelines in July 2018 recommending cautious use of DTG in women of childbearing age and adolescent girls (Update of recommendations on first- and second-line antiretroviral regimens, 2025). This guidance significantly reduced the use of DTG regimens among females, as evidenced by the analysis of reporting trends (Figure 2). However, in recent years, this gender disparity has gradually narrowed (Tiendrebeogo et al., 2024). It is noteworthy that, although the total number of reports from females remains relatively low, a significant risk association has been observed in pregnancy-related SOC signals (*n* = 454; ROR = 14.61, 95% CI = 13.28–16.08) and congenital anomaly signals (*n* = 109; ROR = 8.37, 95% CI = 6.93–10.12), which should not be overlooked.

Adverse effects on both pregnant women and fetuses have been observed with the use of DTG. Despite the wide confidence intervals, our study identified significant associations related to pregnancy (ROR = 22.9) and congenital disorders (ROR = 19.79) within the 0–17 age group and the female subgroup. Research conducted by Molly Hey et al. indicated that women living with HIV (WLHIV) receiving DTG had an increased risk of preterm birth (PTB) and being small for gestational age (SGA) compared to their HIV-negative counterparts (Hey et al., 2025). Ongoing studies have examined the effects of DTG on pregnant women and fetuses (Zash et al., 2018; Zash et al., 2019; CABRERA et al., 2019; van De Ven et al., 2020; Eke et al., 2023; Foster et al., 2023; Gelineau-van Waes et al., 2023; Gill et al., 2023; Kourtis et al., 2023; Saint-Lary et al., 2024). Among the cases of first-trimester DTG exposure reported to the Antiretroviral Pregnancy Registry, the prevalence of birth defects was recorded at 3.3%, with no significant increase in the risk of such defects (Appendix B: Dolutegravir (Tivicay, Tivicay PD) - Safety and Toxicity in Pregnancy NIH, 2025). However, in countries with varying nutritional resource statuses, the weight gain and other metabolic impacts associated with DTG use may adversely affect pregnancy outcomes (Asif et al., 2021; Dontsova et al., 2023).

Significant AEs related to the hepatobiliary system were observed, alongside metabolic effects. Currently, although results from the ADVANCE clinical trial are contradictory (Griesel et al., 2020), numerous studies indicate that DTG is associated with increased weight gain, whether during ART initiation or transition to a DTG-based regimen (Wohl et al., 2019; Calmy et al., 2020; Ando et al., 2021; Jemal, 2024). Women have been identified as a risk factor, potentially due to the inhibition of the melanocortin 4 receptor (MC4R) (Sax et al., 2019; Lake et al., 2020; Jemal, 2024). Higher circulating leptin levels in females stimulate pro-opiomelanocortin (POMC) neurons to produce POMC peptides, which activate the MC4R to suppress food intake and reduce body weight. The expression of leptin receptors on POMC neurons is required for regulating fat distribution in females. Consequently, the inhibitory effect of DTG on MC4R may influence the regulation of female fat distribution (Lake et al., 2020).

Concurrently, abnormal signals related to blood glucose and lipid levels were detected, such as hyperglycemia and hyperlipidemia (see Supplementary Table S2). The weight gain and metabolic effects induced by DTG, including obesity and diabetes, may further contribute to the development of non-alcoholic fatty liver disease (Jemal, 2024; Ram and Subramanian, 2025). These metabolic effects could also potentially lead to the occurrence of pancreatitis (Simeni Njonnou et al., 2020); however, the rarity of related cases complicates this determination (Thompson et al., 2015). In large-scale clinical trials, instances of abnormally elevated liver enzymes and acute liver injury accompanied by jaundice due to hypersensitivity reactions were reported. The immune reconstitution associated with ART treatment may be linked to the occurrence of hepatitis in patients co-infected with hepatitis B virus (HBV) and hepatitis C virus (HCV) (Dolutegravir, 2006). Furthermore, medication errors relating to renal and hepatic dose adjustments in multi-tablet regimens may increase the incidence of these adverse reactions (Kelley et al., 2025). A case report documented a 47-year-old male patient who developed delayed-onset liver injury following the use of Triumeq (abacavir/lamivudine/dolutegravir) (Christensen et al., 2017). However, these findings necessitate further clinical studies for confirmation. Additionally, the shape parameter β of the Weibull distribution is 0.58, and the median time to onset of these DTG-induced DME is 59 days, underscoring the importance of early monitoring of biochemical indicators during medication use.

When administered at a daily dose of 50 mg, DTG results in an approximate 10% reduction in creatinine clearance (CrCl) by inhibiting organic cation transporter 2, which is generally considered clinically insignificant (Koteff et al., 2013). Traditional risk factors such as diabetes and hypertension are prevalent among PLWH, and elderly patients experience ongoing nephron aging, increased drug elimination, and exposure, often necessitating the use of multiple medications that may have potential adverse effects and are prone to interactions (EACS Guidelines, 2024, n.d.). Current research has yet to elucidate the renal failure signals observed in the population aged over 65 years; thus, caution is warranted to prevent overlooking renal injury attributed to the perceived reduction in estimated glomerular filtration rate (eGFR) caused by DTG. We noted a median onset time of 154 days for AEs in elderly patients, suggesting the potential for cumulative toxicity from long-term medication use, thereby underscoring the necessity for long-term monitoring.

The potential risks associated with progressive multifocal leukoencephalopathy (PML) and sensorineural deafness are considerable. PML is a rare and destructive central nervous system (CNS) disease caused by the reactivation of the John Cunningham virus (JCV) in immunocompromised patients (Tan and Koralnik, 2010), a relatively common complication of HIV disease. Moreover, the immune reconstitution inflammatory syndrome (IRIS), which is often induced following the initiation of combination antiretroviral therapy (cART) in PLWH, tends to exacerbate PML (Müller et al., 2010; Fournier et al., 2017). In the study conducted by Ingeborg E. A. Wijting et al., DTG was found to be associated with an increased risk of IRIS (odds ratio [OR] 4.08, 95% CI: 0.99–16.82, *p* = 0.052) (Wijting et al., 2019). The proportion of males affected is notably higher, at 56% (Fournier et al., 2017), which correlates with the IRIS signals we observed and the PML signals identified within the male subgroup. Additionally, DTG-based ART may elevate mitochondrial oxidative stress through various pathways (Mohan et al., 2023), leading to increased production of reactive oxygen species (ROS), oxidative stress, and inflammation, all of which could potentially accelerate the progression of sensorineural deafness. Alarmingly, PML and sensorineural deafness accounted for 66.67% (4 out of 6 cases) and 66.67% (2 out of 3 cases) of fatal outcomes, respectively, in adverse event reports for the 18–45 age group. Notably, PML resulted in a mortality rate of 41.67% (10 out of 24 cases) across all populations. We believe this represents a significant direction for future research.

It is essential to acknowledge the limitations of our study. First, the FAERS database consists of spontaneously submitted reports. While we standardized adverse events using MedDRA terminology and endeavored to collect drug names as comprehensively as possible, the inherent underreporting and reporting biases associated with spontaneous reporting systems may compromise the reliability of the signals. Additionally, due to the absence of exposure data in the reports, the incidence of adverse events cannot be calculated. Second, confounding factors introduced by other ART drugs used in DTG-based combination therapies are challenging to exclude, particularly in the context of fixed-dose combinations. Consequently, while we conducted statistical analyses on combination therapies, the interpretative value of these references is limited. Moreover, the disproportionality analysis method cannot accurately assess the true risk of AEs; it is important to recognize that this method only illustrates a statistical association. Although we also employed a clinically prioritized comprehensive assessment, this potential association should be interpreted cautiously. Finally, further prospective studies are necessary to comprehensively evaluate the results from pharmacovigilance analyses, thereby enhancing and optimizing the rational use of clinical drugs.

## 5 Conclusion

Utilizing large-scale real-world data, our pharmacovigilance study conducted a comprehensive exploration of potential adverse reaction signals associated with the use of DTG. We identified that the use of DTG is associated with AEs related to pregnancy, puerperium and perinatal conditions, hepatobiliary disorders, as well as congenital, familial, and genetic disorders. Although no high clinical priority AE signals were identified overall, subgroup analyses revealed that progressive multifocal leukoencephalopathy in males, progressive multifocal leukoencephalopathy and deafness neurosensory in the 18–45 age group, and hepatic necrosis in the 46–65 age group were rated as high clinical priority and warrant attention. Furthermore, the analysis of the time to AE onset suggested an early failure pattern, with the majority of AEs occurring after 70 days. Therefore, the long-term safety of DTG necessitates a reassessment of the risk-benefit ratio, along with the implementation of structured risk management strategies, including restrictions on contraindicated populations, enhanced monitoring of high-risk subgroups, and post-marketing prospective cohort studies, to mitigate the risk of irreversible organic damage and ensure the sustainability of antiviral therapy.

## Data Availability

Publicly available datasets were analyzed in this study. This data can be found here: https://fis.fda.gov/extensions/FPD-QDE-FAERS/FPD-QDE-FAERS.html.

## Abbreviations

AE, adverse event; ART, antiretroviral therapy; BCPNN, Bayesian Confidence Propagation Neural Network; BIC/FTC/TAF, Bictegravir/Emtricitabine/Tenofovir Alafenamide; cART, combination antiretroviral therapy; CI, confidence Interval; CNS, central nervous system; CrCl, creatinine clearance; DME, Designated Medical Event; DTG, dolutegravir; EBGM, Empirical Bayesian Geometric Mean; eGFR, estimated glomerular filtration rate; FAERS, Food and Drug Administration Adverse Event Reporting System; HBV, hepatitis B virus; HCV, hepatitis C virus; IME, Important Medical Event; INSTI, integrase strand transfer inhibitor; INTIs, integrase inhibitors; IQR, interquartile range; IRIS, immune reconstitution inflammatory syndrome; JCV, John Cunningham virus; MC4R, melanocortin 4 receptor; MedDRA, Medical Dictionary for Regulatory Activities; NTD, neural tube defects; PLWH people living with HIV; PML, progressive multifocal leukoencephalopathy; POMC, pro-opiomelanocortin; PRR, proportional reporting ratio; PS, primary suspected drug; PT, preferred term; PTB, preterm birth; ROR, reporting odds ratio; SGA, small for gestational age; SMQs, standard MedDRA analytical queries; SOC, system organ class; UNAIDS, Joint United Nations Programme on HIV/AIDS; WHO, World Health Organization; WLHIV, women living with HIV.

## References

[B1] Ando N. Nishijima T. Mizushima D. Inaba Y. Kawasaki Y. Kikuchi Y. (2021). Long-term weight gain after initiating combination antiretroviral therapy in treatment-naïve Asian people living with human immunodeficiency virus. Int. J. Infect. Dis. 110, 21–28. 10.1016/j.ijid.2021.07.030 34273516

[B2] Appendix B: Dolutegravir (Tivicay, Tivicay PD)-Safety and Toxicity in Pregnancy NIH (2025). Available online at: https://clinicalinfo.hiv.gov/en/guidelines/perinatal/safety-toxicity-arv-agents-integrase-inhibitors-dolutegravir-tivicay (Accessed April 27, 2025).

[B3] Asif S. Baxevanidi E. Hill A. Venter W. D. F. Fairlie L. Masenya M. (2021). The predicted risk of adverse pregnancy outcomes as a result of treatment-associated obesity in a hypothetical population receiving tenofovir alafenamide/emtricitabine/dolutegravir, tenofovir disoproxil fumarate/emtricitabine/dolutegravir or tenofovir disoproxil fumarate/emtricitabine/efavirenz. AIDS 35, S117–S125. 10.1097/QAD.0000000000003020 34261099

[B4] Brennan A. T. Nattey C. Kileel E. M. Rosen S. Maskew M. Stokes A. C. (2023). Change in body weight and risk of hypertension after switching from efavirenz to dolutegravir in adults living with HIV: evidence from routine care in Johannesburg, South Africa. EClinicalMedicine 57, 101836. 10.1016/j.eclinm.2023.101836 36816348 PMC9932660

[B5] Cabrera R. M. Souder J. P. Steele J. W. Yeo L. Tukeman G. Gorelick D. A. (2019). The antagonism of folate receptor by dolutegravir: developmental toxicity reduction by supplemental folic acid. AIDS 33, 1967–1976. 10.1097/QAD.0000000000002289 31259764 PMC6774845

[B6] Cahn P. Pozniak A. L. Mingrone H. Shuldyakov A. Brites C. Andrade-Villanueva J. F. (2013). Dolutegravir versus raltegravir in antiretroviral-experienced, integrase-inhibitor-naive adults with HIV: week 48 results from the randomised, double-blind, non-inferiority SAILING study. Lancet 382, 700–708. 10.1016/S0140-6736(13)61221-0 23830355

[B7] Calmy A. Tovar Sanchez T. Kouanfack C. Mpoudi-Etame M. Leroy S. Perrineau S. (2020). Dolutegravir-based and low-dose efavirenz-based regimen for the initial treatment of HIV-1 infection (NAMSAL): week 96 results from a two-group, multicentre, randomised, open label, phase 3 non-inferiority trial in Cameroon. Lancet HIV 7, e677–e687. 10.1016/S2352-3018(20)30238-1 33010241

[B8] Castagna A. Maggiolo F. Penco G. Wright D. Mills A. Grossberg R. (2014). Dolutegravir in antiretroviral-experienced patients with raltegravir- and/or elvitegravir-resistant HIV-1: 24-week results of the phase III VIKING-3 study. J. Infect. Dis. 210, 354–362. 10.1093/infdis/jiu051 24446523 PMC4091579

[B9] Cecco S. Puligheddu S. Fusaroli M. Gerratana L. Yan M. Zamagni C. (2024). Emerging toxicities of antibody-drug conjugates for breast cancer: clinical prioritization of adverse events from the FDA adverse event reporting system. Target Oncol. 19, 435–445. 10.1007/s11523-024-01058-9 38696126 PMC11111510

[B10] Chanie G. S. Belachew E. A. Seid A. M. Limenh L. W. Mitku M. L. Beyna A. T. (2025). Patients reported neuropsychiatric adverse events and associated factors among PLHIV patients receiving DTG-based regimen antiretroviral therapy real-life clinical practice in Ethiopia: multi center crossetional study. BMC Psychiatry 25, 383. 10.1186/s12888-025-06820-5 40241041 PMC12004852

[B11] Christensen E. S. Jain R. Roxby A. C. (2017). Abacavir/Dolutegravir/Lamivudine (Triumeq)–induced liver toxicity in a human immunodeficiency virus–infected patient. Open Forum Infect. Dis. 4, ofx122. 10.1093/ofid/ofx122 28748198 PMC5522577

[B12] Cordova E. Hernandez Rendon J. Mingrone V. Martin P. Arevalo Calderon G. Seleme S. (2025). Efficacy of dolutegravir plus lamivudine in treatment-naive people living with HIV without baseline drug-resistance testing available (D2ARLING): 48-week results of a phase 4, randomised, open-label, non-inferiority trial. Lancet HIV 12, e95–e104. 10.1016/S2352-3018(24)00294-7 39826566

[B13] Cutshaw M. K. Harding M. Davenport C. A. Okeke N. L. (2025). Preswitch regimens associated with weight gain among persons with HIV who switch to integrase inhibitor-containing regimens. Open Forum Infect. Dis. 12, ofae752. 10.1093/ofid/ofae752 40124198 PMC11927775

[B14] Data on the HIV Response (2025). Available online at: https://www.who.int/data/gho/data/themes/hiv-aids/data-on-the-hiv-aids-response (Accessed April 20, 2025).

[B15] Dolutegravir (2006). Drugs and lactation database (LactMed®) (Bethesda (MD): National Institute of Child Health and Human Development). Available online at: http://www.ncbi.nlm.nih.gov/books/NBK500631/ (Accessed April 20, 2025).

[B16] Dontsova V. Mohan H. Blanco C. Jao J. Greene N. D. E. Copp A. J. (2023). Metabolic implications and safety of dolutegravir use in pregnancy. Lancet HIV 10, e606–e616. 10.1016/S2352-3018(23)00141-8 37549681 PMC11100098

[B17] EACS Guidelines 2024 (n.d.). EACS Guidelines. Available online at: https://eacs.sanfordguide.com (Accessed April 20, 2025).

[B18] Ebasone P. V. Peer N. Dzudie A. Foaleng M. Melpsa J. Kengne A. P. (2025). A systematic review of mediation analysis frameworks in studies examining the determinants of cardiometabolic outcomes in people living with HIV. BMC Med. Res. Methodol. 25, 41. 10.1186/s12874-025-02498-1 39979870 PMC11844112

[B19] Eke A. C. Mirochnick M. Lockman S. (2023). Antiretroviral therapy and adverse pregnancy outcomes in people living with HIV. N. Engl. J. Med. 388, 344–356. 10.1056/NEJMra2212877 36720135 PMC10400304

[B20] Foster E. G. Palermo N. Y. Liu Y. Edagwa B. Gendelman H. E. Bade A. N. (2023). Inhibition of matrix metalloproteinases by HIV-1 integrase strand transfer inhibitors. Front. Toxicol. 5, 1113032. 10.3389/ftox.2023.1113032 36896351 PMC9988942

[B21] Fournier A. Martin-Blondel G. Lechapt-Zalcman E. Dina J. Kazemi A. Verdon R. (2017). Immune reconstitution inflammatory syndrome unmasking or worsening AIDS-related progressive multifocal leukoencephalopathy: a literature review. Front. Immunol. 8, 577. 10.3389/fimmu.2017.00577 28588577 PMC5440580

[B22] Gandhi R. T. Bedimo R. Hoy J. F. Landovitz R. J. Smith D. M. Eaton E. F. (2023). Antiretroviral drugs for treatment and prevention of HIV infection in adults: 2022 recommendations of the international antiviral society-USA panel. JAMA 329, 63–84. 10.1001/jama.2022.22246 36454551

[B23] Gelineau-van Waes J. van Waes M. A. Hallgren J. Hulen J. Bredehoeft M. Ashley-Koch A. E. (2023). Gene-nutrient interactions that impact magnesium homeostasis increase risk for neural tube defects in mice exposed to dolutegravir. Front. Cell Dev. Biol. 11, 1175917. 10.3389/fcell.2023.1175917 37377737 PMC10292217

[B24] Gill M. M. Khumalo P. Chouraya C. Kunene M. Dlamini F. Hoffman H. J. (2023). Strengthening the evidence: similar rates of neural tube defects among deliveries regardless of maternal HIV status and dolutegravir exposure in hospital birth surveillance in Eswatini. Open Forum Infect. Dis. 10, ofad441. 10.1093/ofid/ofad441 37720700 PMC10502921

[B25] Global HIV & AIDS statistics—Fact sheet UNAIDS (2025). Available online at: https://www.unaids.org/en/resources/fact-sheet (Accessed April 20, 2025).

[B26] Griesel R. Maartens G. Chirehwa M. Sokhela S. Akpomiemie G. Moorhouse M. (2020). CYP2B6 genotype and weight gain differences between dolutegravir and efavirenz. Clin. Infect. Dis. 73, e3902–e3909. 10.1093/cid/ciaa1073 PMC8653639 32960272

[B27] He L. Li J. Cheng X. Luo L. Huang Y. (2025). Association between GLP-1 RAs and DPP-4 inhibitors with biliary disorders: pharmacovigilance analysis. Front. Pharmacol. 16, 1509561. 10.3389/fphar.2025.1509561 40041492 PMC11878242

[B28] Hey M. Thompson L. Portwood C. Sexton H. Kumarendran M. Brandon Z. (2025). Adverse perinatal outcomes associated with different classes of antiretroviral drugs in pregnant women with HIV. AIDS 39, 162–174. 10.1097/QAD.0000000000004032 39407417 PMC11676599

[B29] Hoffmann C. Llibre J. M. (2019). Neuropsychiatric adverse events with dolutegravir and other integrase strand transfer inhibitors. AIDS Rev. 21, 4–10. 10.24875/AIDSRev.19000023 30899113

[B30] Jemal M. (2024). A review of dolutegravir-associated weight gain and secondary metabolic comorbidities. SAGE Open Med. 12, 20503121241260613. 10.1177/20503121241260613 38881592 PMC11179510

[B31] Kelley D. Blackmon K. Nguyen B. L. Rose D. T. (2025). Comparison of error incidence between single-tablet versus multiple-tablet INSTI-based regimens in the inpatient setting. Ann. Pharmacother., 10600280251324337. 10.1177/10600280251324337 40119536 PMC12450241

[B32] Koteff J. Borland J. Chen S. Song I. Peppercorn A. Koshiba T. (2013). A phase 1 study to evaluate the effect of dolutegravir on renal function via measurement of iohexol and para-aminohippurate clearance in healthy subjects. Br. J. Clin. Pharmacol. 75, 990–996. 10.1111/j.1365-2125.2012.04440.x 22905856 PMC3612717

[B33] Kourtis A. P. Zhu W. Lampe M. A. Huang Y.-L. A. Hoover K. W. (2023). Dolutegravir and pregnancy outcomes including neural tube defects in the USA during 2008-20: a national cohort study. Lancet HIV 10, e588–e596. 10.1016/S2352-3018(23)00108-X 37506721 PMC10614030

[B34] Lake J. E. Wu K. Bares S. H. Debroy P. Godfrey C. Koethe J. R. (2020). Risk factors for weight gain following switch to integrase inhibitor–based antiretroviral therapy. Clin. Infect. Dis. 71, e471–e477. 10.1093/cid/ciaa177 32099991 PMC7713693

[B35] Mehta R. Lagishetty C. V. Angelis K. Aylott A. Kahl L. Blair L. (2023). Pharmacokinetic and pharmacokinetic/pharmacodynamic characterization of the dolutegravir/rilpivirine two-drug regimen in SWORD-1/-2 phase 3 studies. Br. J. Clin. Pharmacol. 89, 2190–2200. 10.1111/bcp.15683 36740580

[B36] Mohan J. Ghazi T. Sibiya T. Chuturgoon A. A. (2023). Antiretrovirals promote metabolic syndrome through mitochondrial stress and dysfunction: an *in vitro* study. Biol. (Basel) 12, 580. 10.3390/biology12040580 PMC10135454 37106780

[B37] Müller M. Wandel S. Colebunders R. Attia S. Furrer H. Egger M. (2010). Immune reconstitution inflammatory syndrome in patients starting antiretroviral therapy for HIV infection: a systematic review and meta-analysis. Lancet Infect. Dis. 10, 251–261. 10.1016/S1473-3099(10)70026-8 20334848 PMC4183458

[B38] Palella F. J. Hou Q. Li J. Mahnken J. Carlson K. J. Durham M. (2023). Weight gain and metabolic effects in persons with HIV who switch to ART regimens containing integrase inhibitors or tenofovir alafenamide. J. Acquir Immune Defic. Syndr. 92, 67–75. 10.1097/QAI.0000000000003101 36150045 PMC11706360

[B39] Pérez-Valero I. Corona D. Martínez N. López-Cavanillas M. Lluis C. Luque I. (2023). Real-world discontinuations due to neuropsychiatric symptoms in people living with HIV treated with second-generation integrase inhibitors: a systematic review. Expert Rev. Anti Infect. Ther. 21, 655–665. 10.1080/14787210.2023.2203914 37074798

[B40] Préta L.-H. Chroboczek T. Treluyer J.-M. Chouchana L. (2023). Association of depression and suicidal behaviour reporting with HIV integrase inhibitors: a global pharmacovigilance study. J. Antimicrob. Chemother. 78, 1944–1947. 10.1093/jac/dkad187 37311223

[B41] Raffi F. Jaeger H. Quiros-Roldan E. Albrecht H. Belonosova E. Gatell J. M. (2013). Once-daily dolutegravir versus twice-daily raltegravir in antiretroviral-naive adults with HIV-1 infection (SPRING-2 study): 96 week results from a randomised, double-blind, non-inferiority trial. Lancet Infect. Dis. 13, 927–935. 10.1016/S1473-3099(13)70257-3 24074642

[B42] Ram R. Subramanian A. K R. (2025). Metabolic dysfunction-associated steatotic liver disease (MASLD) in people living with HIV attending centre of excellence in HIV care at a tertiary level teaching hospital in north India—a pilot study. J. Int. Assoc. Provid. AIDS Care 24, 23259582241311912. 10.1177/23259582241311912 39801172 PMC11726528

[B43] Saint-Lary L. Lacroix I. Leroy V. Sommet A. (2024). Integrase inhibitor drugs during pregnancy and congenital anomalies: a case/non-case study from the global pharmacovigilance database VigiBase®. Pharmacol. Res. Perspect. 12, e1247. 10.1002/prp2.1247 39086081 PMC11291555

[B44] Sax P. E. Erlandson K. M. Lake J. E. Mccomsey G. A. Orkin C. Esser S. (2019). Weight gain following initiation of antiretroviral therapy: risk factors in randomized comparative clinical trials. Clin. Infect. Dis. 71, 1379–1389. 10.1093/cid/ciz999 PMC7486849 31606734

[B45] Simeni Njonnou S. R. Henrard S. Noure L. Goffard J.-C. (2020). Severe rhabdomyolysis and acute asymptomatic pancreatitis following the concomitant use of Biktarvy in the setting of hyperosmolar diabetic crisis. BMJ Case Rep. 13, e234483. 10.1136/bcr-2020-234483 PMC7332188 32611654

[B46] Swinkels H. M. Nguyen A. D. Gulick P. G. (2024). HIV and AIDS, in StatPearls. StatPearls Publishing. Available online at: https://www.ncbi.nlm.nih.gov/books/NBK534860/ (Accessed April 20, 2025). 30521281

[B47] Tan C. S. Koralnik I. J. (2010). Progressive multifocal leukoencephalopathy and other disorders caused by JC virus: clinical features and pathogenesis. Lancet Neurol. 9, 425–437. 10.1016/S1474-4422(10)70040-5 20298966 PMC2880524

[B48] Thompson A. B. Wynn B. A. O Akerele D. A Rostad C. Anderson E. J. Camacho-Gonzalez A. F. (2015). Acute pancreatitis associated with dolutegravir and lamivudine/abacavir administration. AIDS 29, 390–392. 10.1097/QAD.0000000000000542 25686687

[B49] Tiendrebeogo T. Malateste K. Poda A. Minga A. Messou E. Chenal H. (2024). Sex-based disparities in the transition to dolutegravir-based antiretroviral therapy in west African HIV cohorts. Open Forum Infect. Dis. 11, ofae139. 10.1093/ofid/ofae139 38680609 PMC11055209

[B50] Update of recommendations on first- and second-line antiretroviral regimens (2025). Available online at: https://www.who.int/publications/i/item/WHO-CDS-HIV-19.15 (Accessed April 27, 2025).

[B51] van De Ven N. S. Pozniak A. L. Levi J. A. Clayden P. Garratt A. Redd C. (2020). Analysis of pharmacovigilance databases for dolutegravir safety in pregnancy. Clin. Infect. Dis. 70, 2599–2606. 10.1093/cid/ciz684 31595301

[B52] Walmsley S. L. Antela A. Clumeck N. Duiculescu D. Eberhard A. Gutiérrez F. (2013). Dolutegravir plus abacavir-lamivudine for the treatment of HIV-1 infection. N. Engl. J. Med. 369, 1807–1818. 10.1056/NEJMoa1215541 24195548

[B53] What’s New (2024). Adult and Adolescent ARV HIV Clinical Guidelines | NIH. Available online at: https://clinicalinfo.hiv.gov/en/guidelines/hiv-clinical-guidelines-adult-and-adolescent-arv/whats-new (Accessed April 20, 2025).

[B54] White E. Kityo C. Spyer M. J. Mujuru H. A. Nankya I. Kekitiinwa A. R. (2025). Virological outcomes and genotypic resistance on dolutegravir-based antiretroviral therapy versus standard of care in children and adolescents: a secondary analysis of the ODYSSEY trial. Lancet HIV 12, e201–e213. 10.1016/S2352-3018(24)00155-3 39978387

[B55] Wijting I. E. A. Wit F. W. N. M. Rokx C. Leyten E. M. S. Lowe S. H. Brinkman K. (2019). Immune reconstitution inflammatory syndrome in HIV infected late presenters starting integrase inhibitor containing antiretroviral therapy. EClinicalMedicine 17, 100210. 10.1016/j.eclinm.2019.11.003 31891143 PMC6933261

[B56] Wohl D. A. Yazdanpanah Y. Baumgarten A. Clarke A. Thompson M. A. Brinson C. (2019). Bictegravir combined with emtricitabine and tenofovir alafenamide versus dolutegravir, abacavir, and lamivudine for initial treatment of HIV-1 infection: week 96 results from a randomised, double-blind, multicentre, phase 3, non-inferiority trial. Lancet HIV 6, e355–e363. 10.1016/S2352-3018(19)30077-3 31068270

[B57] Wu K. Koethe J. Hulgan T. Brown T. Bares S. H. Tassiopoulos K. (2024). Pharmacogenetics of weight gain following switch from efavirenz- to integrase inhibitor-containing regimens. Pharmacogenet Genomics 34, 25–32. 10.1097/FPC.0000000000000515 37910437 PMC10732300

[B58] Zash R. Holmes L. Diseko M. Jacobson D. L. Brummel S. Mayondi G. (2019). Neural-tube defects and antiretroviral treatment regimens in botswana. N. Engl. J. Med. 381, 827–840. 10.1056/NEJMoa1905230 31329379 PMC6995896

[B59] Zash R. Makhema J. Shapiro R. L. (2018). Neural-tube defects with dolutegravir treatment from the time of conception. N. Engl. J. Med. 379, 979–981. 10.1056/NEJMc1807653 30037297 PMC6550482

