# Supplementary material for: Post-marketing safety concerns with dolutegravir: a pharmacovigilance study based on the FDA adverse event reporting system database
Source: Front Pharmacol. 2025 Jul 30;16:1625601. doi: 10.3389/fphar.2025.1625601 (PMC12343725; doi:10.3389/fphar.2025.1625601)
Supplement: Supplementary file 1 [file Table1.doc]

[**Post-marketing safety concerns with dolutegravir: a pharmacovigilance study based on the FDA Adverse Event Reporting System database**](https://pubmed.ncbi.nlm.nih.gov/39780222/)

Juan Su, ^1, †^ Long He, ^2, †^ and Menglei Wang ^1, *^

^1^ The Second People's Hospital of Meishan City, Meishan, China

^2^ School of Pharmacy, Southwest Medical University, Luzhou, China

^†^ These authors contributed equally and share first authorship

^*^ Correspondence: Menglei Wang, [2865046315@qq.com](mailto:2865046315@qq.com)

Supplementary table 1. Four methods of disproportionality analysis.

| Algorithms | Equation | Criteria |
| --- | --- | --- |
| Reporting odds ratio (ROR) | *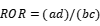*  *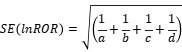*  *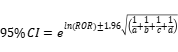* | 95%CI >1, a ≥3 |
| Proportional reporting ratio (PRR) | *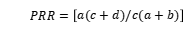*  *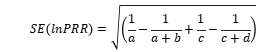*  *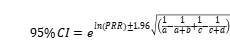* | PRR ≥2, with 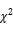≥4 and a ≥3 |
| Bayesian confidence propagation neural network (BCPNN) | *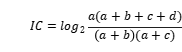*  *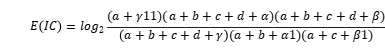*  *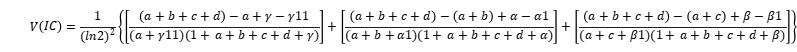*  *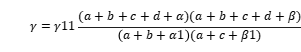*  *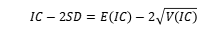*  *α=β=2, α1=β1=γ11=1* | IC025 (lower limit of 95% CI) >0 |
| Empirical Bayesian Geometric Mean (EBGM) | *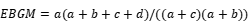*  *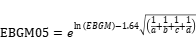* | EBGM05 (lower limit of 95% CI) >2 |

For the above formulas, acquiring values for variables a, b, c, and d was indispensable. Variable “a” stood for those experiencing desired AEs post target drug, “b” represented individuals with non-target AEs post target drug, “c” denoted individuals experiencing the target AE without target drug, and “d” stood for those with non-target AEs without target drug. The total count N was the sum of a, b, c, and d (N=a+b+c+d).

Supplementary table 2

| PT | Case number | ROR(95%Cl) | PRR(Chi-squared) | EBGM(EBGM05) | IC(IC025) | Score | Priority level |
| --- | --- | --- | --- | --- | --- | --- | --- |
| Product dose omission issue | 2014 | 13.76 ( 13.15 - 14.4 ) | 12.97 ( 22139.22 ) | 12.85 ( 12.38 ) | 3.68 ( 3.62 ) | 3 | Moderate |
| Pathogen resistance | 512 | 100.99 ( 92.25 - 110.55 ) | 99.4 ( 46402.14 ) | 92.53 ( 85.79 ) | 6.53 ( 6.4 ) | 3 | Moderate |
| Virologic failure | 454 | 142.41 ( 129.2 - 156.97 ) | 140.43 ( 56831.29 ) | 127.06 ( 117.12 ) | 6.99 ( 6.85 ) | 3 | Moderate |
| Viral mutation identified | 442 | 215.73 ( 195 - 238.67 ) | 212.8 ( 80284.85 ) | 183.48 ( 168.61 ) | 7.52 ( 7.37 ) | 3 | Moderate |
| Exposure during pregnancy | 426 | 11.39 ( 10.35 - 12.54 ) | 11.26 ( 3952.67 ) | 11.17 ( 10.31 ) | 3.48 ( 3.34 ) | 3 | Moderate |
| Product use issue | 341 | 2.79 ( 2.51 - 3.11 ) | 2.77 ( 387.78 ) | 2.77 ( 2.53 ) | 1.47 ( 1.31 ) | 3 | Moderate |
| Foetal exposure during pregnancy | 332 | 6.67 ( 5.99 - 7.44 ) | 6.62 ( 1577.1 ) | 6.59 ( 6.02 ) | 2.72 ( 2.56 ) | 3 | Moderate |
| Blood hiv rna increased | 313 | 393.79 ( 346.99 - 446.9 ) | 389.99 ( 93821.22 ) | 301.51 ( 271.22 ) | 8.24 ( 8.05 ) | 2 | Low |
| Insomnia | 312 | 2.31 ( 2.06 - 2.58 ) | 2.29 ( 228 ) | 2.29 ( 2.09 ) | 1.2 ( 1.03 ) | 2 | Low |
| Treatment noncompliance | 297 | 10.89 ( 9.71 - 12.21 ) | 10.8 ( 2621.67 ) | 10.72 ( 9.74 ) | 3.42 ( 3.25 ) | 2 | Low |
| Inappropriate schedule of product administration | 263 | 2.49 ( 2.21 - 2.81 ) | 2.48 ( 232.43 ) | 2.48 ( 2.24 ) | 1.31 ( 1.13 ) | 2 | Low |
| Product dose omission in error | 261 | 20.14 ( 17.81 - 22.77 ) | 19.98 ( 4638.34 ) | 19.7 ( 17.78 ) | 4.3 ( 4.12 ) | 2 | Low |
| Diabetes mellitus | 229 | 6.66 ( 5.85 - 7.59 ) | 6.62 ( 1088.33 ) | 6.59 ( 5.91 ) | 2.72 ( 2.53 ) | 3 | Moderate |
| Abortion spontaneous | 206 | 10.99 ( 9.57 - 12.61 ) | 10.92 ( 1842.87 ) | 10.84 ( 9.66 ) | 3.44 ( 3.24 ) | 3 | Moderate |
| Immune reconstitution inflammatory syndrome | 203 | 65.95 ( 57.26 - 75.97 ) | 65.55 ( 12295.77 ) | 62.5 ( 55.53 ) | 5.97 ( 5.76 ) | 3 | Moderate |
| Viral load increased | 193 | 100.25 ( 86.57 - 116.09 ) | 99.66 ( 17532.17 ) | 92.76 ( 82.04 ) | 6.54 ( 6.32 ) | 2 | Low |
| Hyperglycaemia | 177 | 10.46 ( 9.02 - 12.13 ) | 10.41 ( 1494.55 ) | 10.34 ( 9.13 ) | 3.37 ( 3.15 ) | 2 | Low |
| Renal failure | 174 | 2.58 ( 2.22 - 3 ) | 2.57 ( 167.37 ) | 2.57 ( 2.27 ) | 1.36 ( 1.14 ) | 4 | Moderate |
| Treatment failure | 148 | 2.84 ( 2.42 - 3.34 ) | 2.83 ( 175.18 ) | 2.83 ( 2.47 ) | 1.5 ( 1.26 ) | 2 | Low |
| Renal impairment | 144 | 3.22 ( 2.74 - 3.8 ) | 3.21 ( 219.42 ) | 3.21 ( 2.8 ) | 1.68 ( 1.44 ) | 3 | Moderate |
| Drug resistance | 139 | 8.96 ( 7.58 - 10.6 ) | 8.93 ( 972.77 ) | 8.88 ( 7.72 ) | 3.15 ( 2.9 ) | 2 | Low |
| Adverse drug reaction | 132 | 2.56 ( 2.16 - 3.04 ) | 2.55 ( 124.66 ) | 2.55 ( 2.21 ) | 1.35 ( 1.1 ) | 2 | Low |
| Blood creatinine increased | 130 | 4.25 ( 3.57 - 5.04 ) | 4.23 ( 320.17 ) | 4.22 ( 3.65 ) | 2.08 ( 1.82 ) | 2 | Low |
| Intentional dose omission | 128 | 8.98 ( 7.55 - 10.69 ) | 8.95 ( 898.31 ) | 8.9 ( 7.69 ) | 3.15 ( 2.9 ) | 2 | Low |
| Suicidal ideation | 124 | 2.95 ( 2.47 - 3.52 ) | 2.94 ( 159.06 ) | 2.94 ( 2.54 ) | 1.56 ( 1.3 ) | 3 | Moderate |
| Neurotoxicity | 110 | 11.7 ( 9.69 - 14.12 ) | 11.66 ( 1063.41 ) | 11.57 ( 9.89 ) | 3.53 ( 3.26 ) | 3 | Moderate |
| Hepatic enzyme increased | 96 | 2.76 ( 2.26 - 3.37 ) | 2.75 ( 106.95 ) | 2.75 ( 2.32 ) | 1.46 ( 1.16 ) | 2 | Low |
| Live birth | 86 | 18.57 ( 15.01 - 22.98 ) | 18.52 ( 1406.27 ) | 18.28 ( 15.3 ) | 4.19 ( 3.88 ) | 2 | Low |
| Syphilis | 82 | 228.74 ( 180.91 - 289.23 ) | 228.17 ( 15820.89 ) | 194.78 ( 160.07 ) | 7.61 ( 7.27 ) | 3 | Moderate |
| Nephropathy toxic | 80 | 14.41 ( 11.56 - 17.97 ) | 14.38 ( 985.21 ) | 14.23 ( 11.83 ) | 3.83 ( 3.51 ) | 3 | Moderate |
| Drug-induced liver injury | 72 | 4.01 ( 3.18 - 5.05 ) | 4 ( 161.51 ) | 3.99 ( 3.29 ) | 2 ( 1.66 ) | 4 | Moderate |
| Cd4 lymphocytes decreased | 72 | 80.25 ( 63.24 - 101.82 ) | 80.07 ( 5301.58 ) | 75.56 ( 61.91 ) | 6.24 ( 5.89 ) | 2 | Low |
| Mental disorder | 69 | 3.23 ( 2.55 - 4.09 ) | 3.22 ( 105.58 ) | 3.22 ( 2.64 ) | 1.69 ( 1.34 ) | 2 | Low |
| Pancreatitis | 65 | 2.89 ( 2.26 - 3.68 ) | 2.88 ( 79.87 ) | 2.88 ( 2.35 ) | 1.53 ( 1.17 ) | 4 | Moderate |
| Hepatitis b | 61 | 22.09 ( 17.15 - 28.46 ) | 22.05 ( 1205.89 ) | 21.71 ( 17.56 ) | 4.44 ( 4.07 ) | 3 | Moderate |
| Extra dose administered | 61 | 2.66 ( 2.07 - 3.42 ) | 2.66 ( 63.09 ) | 2.66 ( 2.15 ) | 1.41 ( 1.04 ) | 2 | Low |
| Premature baby | 60 | 3.65 ( 2.83 - 4.71 ) | 3.65 ( 115.07 ) | 3.64 ( 2.94 ) | 1.86 ( 1.49 ) | 2 | Low |
| Lymphadenopathy | 58 | 3.29 ( 2.55 - 4.26 ) | 3.29 ( 92.26 ) | 3.28 ( 2.65 ) | 1.72 ( 1.34 ) | 2 | Low |
| Hepatotoxicity | 54 | 4.66 ( 3.57 - 6.09 ) | 4.66 ( 154.6 ) | 4.64 ( 3.71 ) | 2.22 ( 1.83 ) | 3 | Moderate |
| Hepatic function abnormal | 54 | 3.02 ( 2.31 - 3.94 ) | 3.01 ( 72.52 ) | 3.01 ( 2.41 ) | 1.59 ( 1.2 ) | 2 | Low |
| Hepatitis c | 50 | 7.75 ( 5.87 - 10.24 ) | 7.74 ( 291.99 ) | 7.7 ( 6.1 ) | 2.95 ( 2.54 ) | 3 | Moderate |
| Stillbirth | 49 | 21 ( 15.83 - 27.85 ) | 20.97 ( 917.37 ) | 20.66 ( 16.31 ) | 4.37 ( 3.96 ) | 3 | Moderate |
| Nervous system disorder | 48 | 4.6 ( 3.46 - 6.11 ) | 4.59 ( 134.55 ) | 4.58 ( 3.61 ) | 2.2 ( 1.78 ) | 2 | Low |
| Product dispensing error | 48 | 3.55 ( 2.68 - 4.72 ) | 3.55 ( 87.75 ) | 3.54 ( 2.8 ) | 1.83 ( 1.41 ) | 2 | Low |
| Transaminases increased | 46 | 3.99 ( 2.99 - 5.33 ) | 3.99 ( 102.65 ) | 3.98 ( 3.12 ) | 1.99 ( 1.57 ) | 2 | Low |
| Blood creatine phosphokinase increased | 46 | 4.04 ( 3.02 - 5.39 ) | 4.03 ( 104.64 ) | 4.02 ( 3.16 ) | 2.01 ( 1.59 ) | 2 | Low |
| Polyneuropathy | 45 | 7.06 ( 5.27 - 9.47 ) | 7.05 ( 232.59 ) | 7.02 ( 5.49 ) | 2.81 ( 2.38 ) | 3 | Moderate |
| Tuberculosis | 45 | 6.74 ( 5.03 - 9.04 ) | 6.73 ( 218.58 ) | 6.7 ( 5.25 ) | 2.74 ( 2.32 ) | 3 | Moderate |
| Jaundice | 45 | 3.9 ( 2.91 - 5.23 ) | 3.9 ( 96.67 ) | 3.89 ( 3.04 ) | 1.96 ( 1.53 ) | 2 | Low |
| Kaposi's sarcoma | 44 | 26.02 ( 19.3 - 35.07 ) | 25.98 ( 1036.62 ) | 25.5 ( 19.86 ) | 4.67 ( 4.24 ) | 3 | Moderate |
| Hiv infection | 43 | 32.72 ( 24.17 - 44.29 ) | 32.68 ( 1288.61 ) | 31.91 ( 24.77 ) | 5 ( 4.56 ) | 4 | Moderate |
| Abnormal dreams | 42 | 3.83 ( 2.83 - 5.19 ) | 3.83 ( 87.6 ) | 3.82 ( 2.97 ) | 1.93 ( 1.49 ) | 2 | Low |
| Osteonecrosis | 40 | 2.78 ( 2.04 - 3.79 ) | 2.78 ( 45.37 ) | 2.77 ( 2.14 ) | 1.47 ( 1.02 ) | 3 | Moderate |
| Acute myocardial infarction | 39 | 2.82 ( 2.06 - 3.85 ) | 2.81 ( 45.49 ) | 2.81 ( 2.16 ) | 1.49 ( 1.03 ) | 3 | Moderate |
| Congenital umbilical hernia | 39 | 190.14 ( 135.91 - 266 ) | 189.91 ( 6409.63 ) | 166.22 ( 125.51 ) | 7.38 ( 6.89 ) | 3 | Moderate |
| Lymphoma | 38 | 4.69 ( 3.41 - 6.44 ) | 4.68 ( 109.65 ) | 4.67 ( 3.58 ) | 2.22 ( 1.76 ) | 4 | Moderate |
| Psychotic disorder | 38 | 2.89 ( 2.1 - 3.97 ) | 2.88 ( 46.68 ) | 2.88 ( 2.21 ) | 1.53 ( 1.06 ) | 2 | Low |
| Dyslipidaemia | 38 | 15.43 ( 11.21 - 21.25 ) | 15.42 ( 506.39 ) | 15.25 ( 11.67 ) | 3.93 ( 3.47 ) | 2 | Low |
| Foetal death | 35 | 11.06 ( 7.93 - 15.43 ) | 11.05 ( 317.32 ) | 10.97 ( 8.3 ) | 3.46 ( 2.97 ) | 4 | Moderate |
| Liver injury | 35 | 2.87 ( 2.06 - 4 ) | 2.87 ( 42.61 ) | 2.87 ( 2.17 ) | 1.52 ( 1.04 ) | 3 | Moderate |
| Pneumocystis jirovecii pneumonia | 35 | 5.53 ( 3.97 - 7.71 ) | 5.52 ( 129.12 ) | 5.5 ( 4.17 ) | 2.46 ( 1.98 ) | 3 | Moderate |
| Low birth weight baby | 34 | 6.79 ( 4.84 - 9.51 ) | 6.78 ( 166.74 ) | 6.75 ( 5.09 ) | 2.76 ( 2.27 ) | 2 | Low |
| Cholestasis | 32 | 3.44 ( 2.43 - 4.87 ) | 3.44 ( 55.26 ) | 3.43 ( 2.57 ) | 1.78 ( 1.28 ) | 3 | Moderate |
| Blood bilirubin increased | 32 | 2.72 ( 1.92 - 3.85 ) | 2.72 ( 34.77 ) | 2.72 ( 2.03 ) | 1.44 ( 0.94 ) | 2 | Low |
| Product use in unapproved therapeutic environment | 32 | 133.83 ( 93.03 - 192.54 ) | 133.7 ( 3828.28 ) | 121.53 ( 89.65 ) | 6.93 ( 6.4 ) | 2 | Low |
| Glomerular filtration rate decreased | 30 | 4.54 ( 3.17 - 6.5 ) | 4.54 ( 82.56 ) | 4.53 ( 3.35 ) | 2.18 ( 1.66 ) | 2 | Low |
| Birth mark | 29 | 256.3 ( 172.19 - 381.49 ) | 256.07 ( 6174.38 ) | 214.74 ( 153.95 ) | 7.75 ( 7.18 ) | 2 | Low |
| Product communication issue | 28 | 6.08 ( 4.2 - 8.82 ) | 6.08 ( 118.28 ) | 6.06 ( 4.44 ) | 2.6 ( 2.06 ) | 2 | Low |
| Foetal growth restriction | 27 | 7.86 ( 5.38 - 11.47 ) | 7.85 ( 160.48 ) | 7.81 ( 5.69 ) | 2.97 ( 2.42 ) | 4 | Moderate |
| Viraemia | 26 | 31.97 ( 21.66 - 47.18 ) | 31.94 ( 761.05 ) | 31.22 ( 22.54 ) | 4.96 ( 4.4 ) | 2 | Low |
| Atrial septal defect | 26 | 6.51 ( 4.43 - 9.57 ) | 6.5 ( 120.55 ) | 6.48 ( 4.69 ) | 2.7 ( 2.14 ) | 2 | Low |
| Cytomegalovirus infection | 26 | 2.99 ( 2.03 - 4.39 ) | 2.98 ( 34.23 ) | 2.98 ( 2.16 ) | 1.58 ( 1.02 ) | 2 | Low |
| Blood triglycerides increased | 25 | 3.66 ( 2.47 - 5.43 ) | 3.66 ( 48.25 ) | 3.65 ( 2.63 ) | 1.87 ( 1.3 ) | 2 | Low |
| Umbilical hernia | 25 | 9.88 ( 6.66 - 14.64 ) | 9.87 ( 197.81 ) | 9.8 ( 7.05 ) | 3.29 ( 2.73 ) | 2 | Low |
| Progressive multifocal leukoencephalopathy | 24 | 5.4 ( 3.62 - 8.07 ) | 5.4 ( 85.7 ) | 5.38 ( 3.85 ) | 2.43 ( 1.85 ) | 5 | Moderate |
| Hiv viraemia | 24 | 195.16 ( 127.12 - 299.61 ) | 195.02 ( 4037.98 ) | 170.12 ( 118.84 ) | 7.41 ( 6.8 ) | 3 | Moderate |
| Splenomegaly | 23 | 3.87 ( 2.57 - 5.82 ) | 3.86 ( 48.68 ) | 3.86 ( 2.74 ) | 1.95 ( 1.36 ) | 3 | Moderate |
| Hyperuricaemia | 23 | 11.89 ( 7.89 - 17.93 ) | 11.89 ( 227.28 ) | 11.79 ( 8.36 ) | 3.56 ( 2.97 ) | 2 | Low |
| Sexual dysfunction | 21 | 3.68 ( 2.4 - 5.65 ) | 3.68 ( 40.92 ) | 3.67 ( 2.57 ) | 1.88 ( 1.26 ) | 2 | Low |
| Polyuria | 21 | 5.03 ( 3.27 - 7.71 ) | 5.02 ( 67.41 ) | 5.01 ( 3.5 ) | 2.32 ( 1.71 ) | 2 | Low |
| Lipodystrophy acquired | 20 | 18.23 ( 11.72 - 28.34 ) | 18.22 ( 321.06 ) | 17.98 ( 12.43 ) | 4.17 ( 3.53 ) | 3 | Moderate |
| Toxic skin eruption | 20 | 4.25 ( 2.74 - 6.59 ) | 4.25 ( 49.46 ) | 4.23 ( 2.93 ) | 2.08 ( 1.45 ) | 3 | Moderate |
| T-lymphocyte count decreased | 20 | 57.37 ( 36.66 - 89.78 ) | 57.34 ( 1061.16 ) | 55 ( 37.81 ) | 5.78 ( 5.14 ) | 2 | Low |
| Polydipsia | 20 | 11.38 ( 7.32 - 17.67 ) | 11.37 ( 187.54 ) | 11.28 ( 7.8 ) | 3.5 ( 2.86 ) | 2 | Low |
| Drug level decreased | 20 | 3.51 ( 2.26 - 5.45 ) | 3.51 ( 35.83 ) | 3.5 ( 2.43 ) | 1.81 ( 1.18 ) | 2 | Low |
| Hepatitis acute | 19 | 6.45 ( 4.11 - 10.12 ) | 6.45 ( 87.01 ) | 6.42 ( 4.4 ) | 2.68 ( 2.04 ) | 3 | Moderate |
| Hypertriglyceridaemia | 19 | 6.77 ( 4.31 - 10.62 ) | 6.76 ( 92.84 ) | 6.73 ( 4.62 ) | 2.75 ( 2.1 ) | 2 | Low |
| Hepatitis a | 19 | 49.86 ( 31.53 - 78.84 ) | 49.83 ( 876.23 ) | 48.06 ( 32.76 ) | 5.59 ( 4.93 ) | 2 | Low |
| Cytomegalovirus chorioretinitis | 18 | 17.73 ( 11.14 - 28.24 ) | 17.73 ( 280.33 ) | 17.5 ( 11.86 ) | 4.13 ( 3.46 ) | 3 | Moderate |
| Meningitis cryptococcal | 17 | 19.94 ( 12.35 - 32.19 ) | 19.93 ( 301.07 ) | 19.65 ( 13.16 ) | 4.3 ( 3.61 ) | 3 | Moderate |
| Ocular icterus | 17 | 8.88 ( 5.51 - 14.31 ) | 8.88 ( 118.07 ) | 8.83 ( 5.92 ) | 3.14 ( 2.46 ) | 2 | Low |
| Intentional underdose | 17 | 3.45 ( 2.14 - 5.55 ) | 3.45 ( 29.44 ) | 3.44 ( 2.31 ) | 1.78 ( 1.1 ) | 2 | Low |
| Neural tube defect | 16 | 56.24 ( 34.1 - 92.76 ) | 56.21 ( 832.35 ) | 53.96 ( 35.5 ) | 5.75 ( 5.04 ) | 4 | Moderate |
| Immune reconstitution inflammatory syndrome associated tuberculosis | 16 | 83.15 ( 50.17 - 137.81 ) | 83.1 ( 1221.25 ) | 78.26 ( 51.28 ) | 6.29 ( 5.57 ) | 3 | Moderate |
| Ventricular septal defect | 16 | 6.05 ( 3.7 - 9.89 ) | 6.05 ( 67.08 ) | 6.02 ( 3.99 ) | 2.59 ( 1.89 ) | 3 | Moderate |
| Polydactyly | 16 | 29.08 ( 17.72 - 47.73 ) | 29.07 ( 424.36 ) | 28.47 ( 18.81 ) | 4.83 ( 4.12 ) | 2 | Low |
| Mycobacterium avium complex infection | 15 | 11.23 ( 6.75 - 18.67 ) | 11.22 ( 138.53 ) | 11.14 ( 7.28 ) | 3.48 ( 2.75 ) | 3 | Moderate |
| Cachexia | 15 | 5.93 ( 3.57 - 9.85 ) | 5.93 ( 61.22 ) | 5.91 ( 3.86 ) | 2.56 ( 1.84 ) | 3 | Moderate |
| Gestational diabetes | 15 | 5.93 ( 3.57 - 9.84 ) | 5.93 ( 61.14 ) | 5.9 ( 3.86 ) | 2.56 ( 1.84 ) | 3 | Moderate |
| Atypical mycobacterial infection | 15 | 22.85 ( 13.71 - 38.07 ) | 22.84 ( 307.89 ) | 22.47 ( 14.66 ) | 4.49 ( 3.76 ) | 3 | Moderate |
| Hepatitis b reactivation | 15 | 7.03 ( 4.23 - 11.67 ) | 7.02 ( 77.07 ) | 6.99 ( 4.57 ) | 2.81 ( 2.08 ) | 3 | Moderate |
| Psychiatric symptom | 15 | 3.85 ( 2.32 - 6.39 ) | 3.85 ( 31.55 ) | 3.84 ( 2.51 ) | 1.94 ( 1.22 ) | 2 | Low |
| Hyperlipidaemia | 15 | 3.37 ( 2.03 - 5.6 ) | 3.37 ( 24.94 ) | 3.36 ( 2.2 ) | 1.75 ( 1.03 ) | 2 | Low |
| Low density lipoprotein increased | 15 | 4.3 ( 2.59 - 7.13 ) | 4.3 ( 37.81 ) | 4.28 ( 2.8 ) | 2.1 ( 1.38 ) | 2 | Low |
| Patent ductus arteriosus | 14 | 7.77 ( 4.59 - 13.14 ) | 7.77 ( 82.08 ) | 7.73 ( 4.98 ) | 2.95 ( 2.2 ) | 3 | Moderate |
| Abortion | 14 | 9.46 ( 5.59 - 16.01 ) | 9.46 ( 105.18 ) | 9.4 ( 6.05 ) | 3.23 ( 2.48 ) | 3 | Moderate |
| Hepatomegaly | 14 | 3.3 ( 1.95 - 5.58 ) | 3.3 ( 22.42 ) | 3.3 ( 2.13 ) | 1.72 ( 0.97 ) | 2 | Low |
| Blood creatine increased | 14 | 7.03 ( 4.16 - 11.88 ) | 7.02 ( 71.95 ) | 6.99 ( 4.51 ) | 2.81 ( 2.06 ) | 2 | Low |
| Tinea pedis | 14 | 18.13 ( 10.7 - 30.73 ) | 18.13 ( 223.48 ) | 17.89 ( 11.51 ) | 4.16 ( 3.41 ) | 2 | Low |
| Congenital skin dimples | 14 | 90.93 ( 52.91 - 156.3 ) | 90.9 ( 1164.77 ) | 85.12 ( 54.1 ) | 6.41 ( 5.64 ) | 2 | Low |
| Anogenital warts | 14 | 29.12 ( 17.15 - 49.46 ) | 29.11 ( 371.85 ) | 28.51 ( 18.3 ) | 4.83 ( 4.08 ) | 2 | Low |
| Hepatitis cholestatic | 13 | 5.17 ( 3 - 8.92 ) | 5.17 ( 43.59 ) | 5.16 ( 3.27 ) | 2.37 ( 1.59 ) | 3 | Moderate |
| Renal tubular disorder | 13 | 10.48 ( 6.07 - 18.1 ) | 10.48 ( 110.61 ) | 10.41 ( 6.59 ) | 3.38 ( 2.6 ) | 3 | Moderate |
| Psychiatric decompensation | 12 | 11.04 ( 6.25 - 19.49 ) | 11.04 ( 108.63 ) | 10.95 ( 6.81 ) | 3.45 ( 2.65 ) | 2 | Low |
| Pre-eclampsia | 12 | 5.15 ( 2.92 - 9.08 ) | 5.15 ( 39.98 ) | 5.13 ( 3.19 ) | 2.36 ( 1.56 ) | 2 | Low |
| Viral load abnormal | 12 | 133.61 ( 73.79 - 241.94 ) | 133.56 ( 1434.21 ) | 121.42 ( 73.88 ) | 6.92 ( 6.08 ) | 2 | Low |
| Hepatic necrosis | 11 | 9 ( 4.97 - 16.29 ) | 9 ( 77.69 ) | 8.94 ( 5.45 ) | 3.16 ( 2.32 ) | 5 | Moderate |
| Mitochondrial toxicity | 11 | 27.5 ( 15.13 - 49.96 ) | 27.49 ( 275.08 ) | 26.95 ( 16.35 ) | 4.75 ( 3.91 ) | 3 | Moderate |
| Pulmonary tuberculosis | 11 | 5.62 ( 3.11 - 10.16 ) | 5.62 ( 41.57 ) | 5.6 ( 3.41 ) | 2.48 ( 1.65 ) | 3 | Moderate |
| Talipes | 11 | 7.57 ( 4.19 - 13.7 ) | 7.57 ( 62.35 ) | 7.53 ( 4.59 ) | 2.91 ( 2.08 ) | 3 | Moderate |
| Disseminated tuberculosis | 11 | 9.86 ( 5.45 - 17.85 ) | 9.86 ( 86.9 ) | 9.79 ( 5.96 ) | 3.29 ( 2.46 ) | 3 | Moderate |
| Monkeypox | 11 | 77.94 ( 42.42 - 143.18 ) | 77.91 ( 788.77 ) | 73.64 ( 44.27 ) | 6.2 ( 5.34 ) | 3 | Moderate |
| Hiv-associated neurocognitive disorder | 10 | 150.56 ( 78.27 - 289.6 ) | 150.51 ( 1333.62 ) | 135.25 ( 78.24 ) | 7.08 ( 6.16 ) | 3 | Moderate |
| Ectopic pregnancy | 10 | 7.99 ( 4.29 - 14.87 ) | 7.98 ( 60.73 ) | 7.94 ( 4.72 ) | 2.99 ( 2.12 ) | 3 | Moderate |
| Drug screen positive | 10 | 3.41 ( 1.83 - 6.35 ) | 3.41 ( 17.02 ) | 3.41 ( 2.03 ) | 1.77 ( 0.9 ) | 2 | Low |
| Trisomy 21 | 10 | 18.85 ( 10.09 - 35.18 ) | 18.84 ( 166.57 ) | 18.59 ( 11.03 ) | 4.22 ( 3.34 ) | 2 | Low |
| Anencephaly | 9 | 35.38 ( 18.25 - 68.6 ) | 35.37 ( 292.79 ) | 34.48 ( 19.81 ) | 5.11 ( 4.18 ) | 5 | Moderate |
| Spina bifida | 9 | 8.7 ( 4.51 - 16.75 ) | 8.69 ( 60.89 ) | 8.64 ( 4.99 ) | 3.11 ( 2.2 ) | 5 | Moderate |
| Mycobacterial infection | 9 | 9.97 ( 5.17 - 19.21 ) | 9.97 ( 72.06 ) | 9.9 ( 5.72 ) | 3.31 ( 2.39 ) | 4 | Moderate |
| Prostatitis | 9 | 6.01 ( 3.12 - 11.57 ) | 6.01 ( 37.43 ) | 5.99 ( 3.46 ) | 2.58 ( 1.67 ) | 2 | Low |
| Anxiety disorder | 9 | 5.32 ( 2.76 - 10.23 ) | 5.31 ( 31.4 ) | 5.3 ( 3.06 ) | 2.41 ( 1.49 ) | 2 | Low |
| Hepatosplenomegaly | 9 | 8.57 ( 4.45 - 16.5 ) | 8.56 ( 59.74 ) | 8.51 ( 4.92 ) | 3.09 ( 2.17 ) | 2 | Low |
| Genital herpes | 9 | 11.61 ( 6.02 - 22.38 ) | 11.61 ( 86.48 ) | 11.51 ( 6.65 ) | 3.53 ( 2.61 ) | 2 | Low |
| Exposure via breast milk | 9 | 3.68 ( 1.91 - 7.08 ) | 3.68 ( 17.53 ) | 3.67 ( 2.12 ) | 1.88 ( 0.96 ) | 2 | Low |
| Unmasking of previously unidentified disease | 9 | 13.53 ( 7.02 - 26.1 ) | 13.53 ( 103.39 ) | 13.4 ( 7.74 ) | 3.74 ( 2.83 ) | 2 | Low |
| Disseminated mycobacterium avium complex infection | 8 | 66.66 ( 32.76 - 135.62 ) | 66.64 ( 492.47 ) | 63.5 ( 35.05 ) | 5.99 ( 5 ) | 4 | Moderate |
| Acute psychosis | 8 | 6.64 ( 3.31 - 13.29 ) | 6.63 ( 38.1 ) | 6.61 ( 3.69 ) | 2.72 ( 1.76 ) | 3 | Moderate |
| Abortion missed | 8 | 17.04 ( 8.48 - 34.23 ) | 17.04 ( 119.22 ) | 16.83 ( 9.39 ) | 4.07 ( 3.1 ) | 3 | Moderate |
| Gonorrhoea | 8 | 56.37 ( 27.78 - 114.4 ) | 56.36 ( 417.28 ) | 54.1 ( 29.93 ) | 5.76 ( 4.77 ) | 3 | Moderate |
| Coronary artery stenosis | 8 | 4.64 ( 2.32 - 9.3 ) | 4.64 ( 22.79 ) | 4.63 ( 2.59 ) | 2.21 ( 1.25 ) | 3 | Moderate |
| Acute hepatitis c | 8 | 71.61 ( 35.15 - 145.88 ) | 71.59 ( 528.31 ) | 67.97 ( 37.48 ) | 6.09 ( 5.1 ) | 3 | Moderate |
| Polyhydramnios | 8 | 16.28 ( 8.11 - 32.7 ) | 16.28 ( 113.31 ) | 16.09 ( 8.98 ) | 4.01 ( 3.04 ) | 3 | Moderate |
| Hydrops foetalis | 8 | 24.42 ( 12.13 - 49.15 ) | 24.41 ( 176.39 ) | 23.99 ( 13.36 ) | 4.58 ( 3.61 ) | 3 | Moderate |
| Meningomyelocele | 8 | 25.17 ( 12.51 - 50.67 ) | 25.17 ( 182.2 ) | 24.72 ( 13.77 ) | 4.63 ( 3.66 ) | 3 | Moderate |
| Ultrasound foetal abnormal | 8 | 199.97 ( 95.08 - 420.6 ) | 199.92 ( 1375.76 ) | 173.83 ( 93.32 ) | 7.44 ( 6.41 ) | 3 | Moderate |
| Human immunodeficiency virus transmission | 8 | 80.9 ( 39.62 - 165.2 ) | 80.88 ( 594.85 ) | 76.29 ( 41.98 ) | 6.25 ( 5.26 ) | 3 | Moderate |
| Beta 2 microglobulin urine increased | 8 | 158.19 ( 75.98 - 329.32 ) | 158.15 ( 1115.98 ) | 141.39 ( 76.55 ) | 7.14 ( 6.13 ) | 3 | Moderate |
| Lymphadenopathy mediastinal | 8 | 8.29 ( 4.14 - 16.62 ) | 8.29 ( 50.98 ) | 8.25 ( 4.61 ) | 3.04 ( 2.08 ) | 3 | Moderate |
| Lung consolidation | 8 | 6.64 ( 3.32 - 13.31 ) | 6.64 ( 38.16 ) | 6.62 ( 3.7 ) | 2.73 ( 1.76 ) | 2 | Low |
| Depressive symptom | 8 | 4.42 ( 2.21 - 8.86 ) | 4.42 ( 21.12 ) | 4.41 ( 2.47 ) | 2.14 ( 1.18 ) | 2 | Low |
| Creatinine renal clearance decreased | 8 | 4.15 ( 2.07 - 8.3 ) | 4.15 ( 19.04 ) | 4.14 ( 2.31 ) | 2.05 ( 1.08 ) | 2 | Low |
| Granuloma | 8 | 5.06 ( 2.53 - 10.14 ) | 5.06 ( 25.98 ) | 5.05 ( 2.82 ) | 2.34 ( 1.37 ) | 2 | Low |
| Anal cancer | 7 | 13.29 ( 6.31 - 27.97 ) | 13.28 ( 78.72 ) | 13.16 ( 7.06 ) | 3.72 ( 2.69 ) | 3 | Moderate |
| Heart disease congenital | 7 | 4.17 ( 1.99 - 8.76 ) | 4.17 ( 16.83 ) | 4.16 ( 2.24 ) | 2.06 ( 1.04 ) | 3 | Moderate |
| Acute hepatitis b | 7 | 40.14 ( 18.92 - 85.16 ) | 40.14 ( 259.27 ) | 38.98 ( 20.78 ) | 5.28 ( 4.25 ) | 3 | Moderate |
| Abdominal lymphadenopathy | 7 | 12.95 ( 6.15 - 27.27 ) | 12.95 ( 76.44 ) | 12.83 ( 6.88 ) | 3.68 ( 2.66 ) | 3 | Moderate |
| Visceral leishmaniasis | 7 | 18.93 ( 8.97 - 39.91 ) | 18.92 ( 117.15 ) | 18.67 ( 10 ) | 4.22 ( 3.19 ) | 3 | Moderate |
| Lipoatrophy | 7 | 16.38 ( 7.77 - 34.53 ) | 16.38 ( 99.86 ) | 16.19 ( 8.68 ) | 4.02 ( 2.99 ) | 2 | Low |
| Seborrhoeic dermatitis | 7 | 8.49 ( 4.04 - 17.86 ) | 8.49 ( 45.96 ) | 8.44 ( 4.53 ) | 3.08 ( 2.05 ) | 2 | Low |
| Alcohol abuse | 7 | 4.72 ( 2.25 - 9.91 ) | 4.72 ( 20.44 ) | 4.71 ( 2.53 ) | 2.23 ( 1.21 ) | 2 | Low |
| Facial wasting | 7 | 64.85 ( 30.36 - 138.5 ) | 64.83 ( 419.42 ) | 61.86 ( 32.78 ) | 5.95 ( 4.9 ) | 2 | Low |
| Fat redistribution | 7 | 57.6 ( 27.02 - 122.77 ) | 57.59 ( 373.01 ) | 55.23 ( 29.32 ) | 5.79 ( 4.74 ) | 2 | Low |
| Gene mutation | 7 | 5.7 ( 2.71 - 11.98 ) | 5.7 ( 27 ) | 5.68 ( 3.05 ) | 2.51 ( 1.48 ) | 2 | Low |
| Bilirubin conjugated increased | 7 | 7.5 ( 3.57 - 15.76 ) | 7.5 ( 39.18 ) | 7.46 ( 4.01 ) | 2.9 ( 1.88 ) | 2 | Low |
| Helicobacter gastritis | 7 | 10.78 ( 5.12 - 22.69 ) | 10.78 ( 61.61 ) | 10.7 ( 5.74 ) | 3.42 ( 2.39 ) | 2 | Low |
| Body fat disorder | 7 | 23.96 ( 11.35 - 50.6 ) | 23.96 ( 151.26 ) | 23.55 ( 12.6 ) | 4.56 ( 3.53 ) | 2 | Low |
| Lipids increased | 7 | 7.73 ( 3.68 - 16.25 ) | 7.73 ( 40.75 ) | 7.69 ( 4.13 ) | 2.94 ( 1.92 ) | 2 | Low |
| Opportunistic infection | 6 | 6.56 ( 2.94 - 14.63 ) | 6.56 ( 28.12 ) | 6.53 ( 3.34 ) | 2.71 ( 1.61 ) | 4 | Moderate |
| Meningocele | 6 | 82.8 ( 36.29 - 188.9 ) | 82.78 ( 456.24 ) | 77.97 ( 39.1 ) | 6.28 ( 5.16 ) | 4 | Moderate |
| Faeces pale | 6 | 5.52 ( 2.48 - 12.31 ) | 5.52 ( 22.11 ) | 5.5 ( 2.81 ) | 2.46 ( 1.37 ) | 3 | Moderate |
| Maternal drugs affecting foetus | 6 | 3.94 ( 1.77 - 8.77 ) | 3.94 ( 13.1 ) | 3.93 ( 2.01 ) | 1.97 ( 0.88 ) | 3 | Moderate |
| Multimorbidity | 6 | 8.44 ( 3.78 - 18.83 ) | 8.44 ( 39.08 ) | 8.39 ( 4.29 ) | 3.07 ( 1.97 ) | 3 | Moderate |
| Electrocardiogram st segment elevation | 6 | 4.45 ( 2 - 9.92 ) | 4.45 ( 15.99 ) | 4.44 ( 2.27 ) | 2.15 ( 1.06 ) | 3 | Moderate |
| Fanconi syndrome | 6 | 8 ( 3.58 - 17.84 ) | 7.99 ( 36.5 ) | 7.95 ( 4.06 ) | 2.99 ( 1.9 ) | 3 | Moderate |
| Premature separation of placenta | 6 | 8.51 ( 3.81 - 18.99 ) | 8.51 ( 39.5 ) | 8.46 ( 4.32 ) | 3.08 ( 1.99 ) | 3 | Moderate |
| Teratogenicity | 6 | 37.32 ( 16.58 - 84 ) | 37.31 ( 206.21 ) | 36.31 ( 18.42 ) | 5.18 ( 4.07 ) | 3 | Moderate |
| Renal hypoplasia | 6 | 63.08 ( 27.81 - 143.09 ) | 63.07 ( 349.86 ) | 60.25 ( 30.36 ) | 5.91 ( 4.79 ) | 3 | Moderate |
| Sepsis neonatal | 6 | 17.7 ( 7.91 - 39.62 ) | 17.7 ( 93.29 ) | 17.48 ( 8.91 ) | 4.13 ( 3.03 ) | 3 | Moderate |
| Cerebral toxoplasmosis | 6 | 13.68 ( 6.12 - 30.58 ) | 13.68 ( 69.79 ) | 13.55 ( 6.91 ) | 3.76 ( 2.66 ) | 3 | Moderate |
| Microcephaly | 6 | 10.05 ( 4.5 - 22.44 ) | 10.05 ( 48.51 ) | 9.98 ( 5.1 ) | 3.32 ( 2.22 ) | 3 | Moderate |
| Congenital teratoma | 6 | 158.97 ( 68.16 - 370.77 ) | 158.94 ( 840.77 ) | 142.02 ( 69.92 ) | 7.15 ( 5.99 ) | 3 | Moderate |
| Secondary syphilis | 6 | 122.28 ( 52.98 - 282.22 ) | 122.26 ( 660.63 ) | 112.01 ( 55.63 ) | 6.81 ( 5.67 ) | 3 | Moderate |
| Arnold-chiari malformation | 6 | 12.44 ( 5.57 - 27.79 ) | 12.44 ( 62.52 ) | 12.33 ( 6.29 ) | 3.62 ( 2.53 ) | 3 | Moderate |
| High density lipoprotein decreased | 6 | 5.7 ( 2.56 - 12.71 ) | 5.7 ( 23.13 ) | 5.68 ( 2.9 ) | 2.51 ( 1.41 ) | 3 | Moderate |
| Calculus urinary | 6 | 7.69 ( 3.45 - 17.17 ) | 7.69 ( 34.74 ) | 7.65 ( 3.91 ) | 2.94 ( 1.84 ) | 2 | Low |
| Premature rupture of membranes | 6 | 5.35 ( 2.4 - 11.92 ) | 5.34 ( 21.1 ) | 5.33 ( 2.72 ) | 2.41 ( 1.32 ) | 2 | Low |
| Viral load decreased | 6 | 80.29 ( 35.21 - 183.04 ) | 80.27 ( 442.87 ) | 75.74 ( 38.01 ) | 6.24 ( 5.12 ) | 2 | Low |
| Blood hiv rna | 6 | 198.71 ( 84.24 - 468.72 ) | 198.67 ( 1026.15 ) | 172.89 ( 84.32 ) | 7.43 ( 6.26 ) | 2 | Low |
| Drug screen false positive | 6 | 5.08 ( 2.28 - 11.32 ) | 5.08 ( 19.57 ) | 5.06 ( 2.59 ) | 2.34 ( 1.25 ) | 2 | Low |
| Glycosuria | 6 | 10.13 ( 4.53 - 22.61 ) | 10.12 ( 48.96 ) | 10.05 ( 5.13 ) | 3.33 ( 2.23 ) | 2 | Low |
| Hyperlactacidaemia | 6 | 4.49 ( 2.02 - 10.02 ) | 4.49 ( 16.24 ) | 4.48 ( 2.29 ) | 2.16 ( 1.07 ) | 2 | Low |
| Cd4 lymphocytes abnormal | 6 | 48.76 ( 21.59 - 110.15 ) | 48.75 ( 270.68 ) | 47.06 ( 23.8 ) | 5.56 ( 4.44 ) | 2 | Low |
| Cerebral ventricle dilatation | 6 | 12.27 ( 5.49 - 27.41 ) | 12.26 ( 61.5 ) | 12.16 ( 6.21 ) | 3.6 ( 2.51 ) | 2 | Low |
| Hiv test positive | 6 | 20.75 ( 9.26 - 46.49 ) | 20.75 ( 111.05 ) | 20.44 ( 10.41 ) | 4.35 ( 3.25 ) | 2 | Low |
| Cd4 lymphocytes increased | 6 | 59.76 ( 26.37 - 135.43 ) | 59.75 ( 331.65 ) | 57.22 ( 28.86 ) | 5.84 ( 4.72 ) | 2 | Low |
| Vertical infection transmission | 6 | 147.19 ( 63.32 - 342.15 ) | 147.16 ( 783.93 ) | 132.55 ( 65.44 ) | 7.05 ( 5.9 ) | 2 | Low |
| Foetal cardiac disorder | 5 | 44.75 ( 18.35 - 109.12 ) | 44.75 ( 206.85 ) | 43.32 ( 20.55 ) | 5.44 ( 4.24 ) | 5 | Moderate |
| Foetal distress syndrome | 5 | 5.1 ( 2.12 - 12.27 ) | 5.1 ( 16.41 ) | 5.08 ( 2.44 ) | 2.35 ( 1.16 ) | 4 | Moderate |
| Bone infarction | 5 | 23.74 ( 9.8 - 57.49 ) | 23.74 ( 106.98 ) | 23.34 ( 11.13 ) | 4.54 ( 3.35 ) | 3 | Moderate |
| Hyperferritinaemia | 5 | 20.96 ( 8.66 - 50.71 ) | 20.96 ( 93.54 ) | 20.65 ( 9.86 ) | 4.37 ( 3.18 ) | 3 | Moderate |
| Burkitt's lymphoma | 5 | 20.57 ( 8.5 - 49.76 ) | 20.57 ( 91.65 ) | 20.27 ( 9.68 ) | 4.34 ( 3.15 ) | 3 | Moderate |
| Pyelocaliectasis | 5 | 10.45 ( 4.33 - 25.19 ) | 10.45 ( 42.37 ) | 10.37 ( 4.97 ) | 3.37 ( 2.19 ) | 3 | Moderate |
| Renal dysplasia | 5 | 28.55 ( 11.77 - 69.24 ) | 28.54 ( 130.1 ) | 27.96 ( 13.32 ) | 4.81 ( 3.61 ) | 3 | Moderate |
| Neurosyphilis | 5 | 63.08 ( 25.72 - 154.72 ) | 63.07 ( 291.55 ) | 60.25 ( 28.44 ) | 5.91 ( 4.7 ) | 3 | Moderate |
| Encephalocele | 5 | 39.43 ( 16.2 - 95.96 ) | 39.42 ( 181.81 ) | 38.31 ( 18.2 ) | 5.26 ( 4.06 ) | 3 | Moderate |
| Exomphalos | 5 | 23.24 ( 9.6 - 56.27 ) | 23.24 ( 104.56 ) | 22.85 ( 10.9 ) | 4.51 ( 3.32 ) | 3 | Moderate |
| Exophthalmos | 5 | 4.93 ( 2.05 - 11.87 ) | 4.93 ( 15.61 ) | 4.92 ( 2.36 ) | 2.3 ( 1.12 ) | 3 | Moderate |
| Cell death | 5 | 4.61 ( 1.92 - 11.1 ) | 4.61 ( 14.09 ) | 4.6 ( 2.21 ) | 2.2 ( 1.02 ) | 2 | Low |
| Fat tissue increased | 5 | 6.37 ( 2.64 - 15.33 ) | 6.37 ( 22.52 ) | 6.34 ( 3.04 ) | 2.66 ( 1.48 ) | 2 | Low |
| Viral load undetectable | 5 | 135.17 ( 53.86 - 339.27 ) | 135.15 ( 604.15 ) | 122.73 ( 56.83 ) | 6.94 ( 5.7 ) | 2 | Low |
| Fear of death | 5 | 4.87 ( 2.03 - 11.73 ) | 4.87 ( 15.34 ) | 4.86 ( 2.33 ) | 2.28 ( 1.1 ) | 2 | Low |
| Twin pregnancy | 5 | 14.43 ( 5.98 - 34.84 ) | 14.43 ( 61.81 ) | 14.28 ( 6.83 ) | 3.84 ( 2.65 ) | 2 | Low |
| Cryptorchism | 5 | 9.1 ( 3.78 - 21.93 ) | 9.1 ( 35.79 ) | 9.04 ( 4.33 ) | 3.18 ( 1.99 ) | 2 | Low |
| Soliloquy | 5 | 10.65 ( 4.42 - 25.68 ) | 10.65 ( 43.36 ) | 10.57 ( 5.06 ) | 3.4 ( 2.22 ) | 2 | Low |
| Genotype drug resistance test positive | 5 | 7.14 ( 2.96 - 17.19 ) | 7.14 ( 26.24 ) | 7.1 ( 3.4 ) | 2.83 ( 1.65 ) | 2 | Low |
| Ventricular hypertrophy | 5 | 10.91 ( 4.53 - 26.31 ) | 10.91 ( 44.64 ) | 10.83 ( 5.18 ) | 3.44 ( 2.25 ) | 2 | Low |
| Lipids abnormal | 5 | 14.82 ( 6.14 - 35.78 ) | 14.82 ( 63.7 ) | 14.66 ( 7.01 ) | 3.87 ( 2.69 ) | 2 | Low |
| Sickle cell disease | 5 | 15.51 ( 6.42 - 37.46 ) | 15.51 ( 67.08 ) | 15.34 ( 7.34 ) | 3.94 ( 2.75 ) | 2 | Low |
| White matter lesion | 5 | 6.39 ( 2.65 - 15.38 ) | 6.39 ( 22.61 ) | 6.36 ( 3.05 ) | 2.67 ( 1.49 ) | 2 | Low |
| Hepatitis b dna increased | 5 | 12.45 ( 5.16 - 30.04 ) | 12.45 ( 52.15 ) | 12.34 ( 5.91 ) | 3.63 ( 2.44 ) | 2 | Low |
| Hypoplastic nasal cartilage | 5 | 662.34 ( 226.38 - 1937.93 ) | 662.24 ( 2200.81 ) | 441.83 ( 179.94 ) | 8.79 ( 7.41 ) | 2 | Low |
| Cd4/cd8 ratio decreased | 5 | 41.92 ( 17.21 - 102.12 ) | 41.91 ( 193.56 ) | 40.66 ( 19.3 ) | 5.35 ( 4.15 ) | 2 | Low |
| Male sexual dysfunction | 5 | 16.52 ( 6.84 - 39.9 ) | 16.51 ( 71.98 ) | 16.32 ( 7.8 ) | 4.03 ( 2.84 ) | 2 | Low |
| Gastroschisis | 4 | 17.04 ( 6.35 - 45.68 ) | 17.04 ( 59.61 ) | 16.83 ( 7.37 ) | 4.07 ( 2.77 ) | 5 | Moderate |
| Syndactyly | 4 | 9.87 ( 3.69 - 26.39 ) | 9.87 ( 31.63 ) | 9.8 ( 4.3 ) | 3.29 ( 2 ) | 4 | Moderate |
| Congenital central nervous system anomaly | 4 | 8.29 ( 3.1 - 22.16 ) | 8.29 ( 25.49 ) | 8.25 ( 3.62 ) | 3.04 ( 1.75 ) | 4 | Moderate |
| Plasmablastic lymphoma | 4 | 23.65 ( 8.8 - 63.58 ) | 23.65 ( 85.25 ) | 23.25 ( 10.17 ) | 4.54 ( 3.23 ) | 4 | Moderate |
| Acute hiv infection | 4 | 24.53 ( 9.12 - 65.96 ) | 24.53 ( 88.63 ) | 24.1 ( 10.53 ) | 4.59 ( 3.29 ) | 3 | Moderate |
| Hepatitis viral | 4 | 12.74 ( 4.76 - 34.1 ) | 12.74 ( 42.84 ) | 12.62 ( 5.54 ) | 3.66 ( 2.36 ) | 3 | Moderate |
| Necrotising retinitis | 4 | 10.92 ( 4.08 - 29.23 ) | 10.92 ( 35.77 ) | 10.84 ( 4.76 ) | 3.44 ( 2.14 ) | 3 | Moderate |
| Prurigo | 4 | 13.31 ( 4.97 - 35.65 ) | 13.31 ( 45.09 ) | 13.19 ( 5.78 ) | 3.72 ( 2.42 ) | 3 | Moderate |
| Histoplasmosis disseminated | 4 | 5.16 ( 1.93 - 13.77 ) | 5.16 ( 13.36 ) | 5.14 ( 2.26 ) | 2.36 ( 1.07 ) | 3 | Moderate |
| Pancreatic failure | 4 | 4.67 ( 1.75 - 12.47 ) | 4.67 ( 11.5 ) | 4.66 ( 2.05 ) | 2.22 ( 0.93 ) | 3 | Moderate |
| Urethritis gonococcal | 4 | 1059.72 ( 284.55 - 3946.61 ) | 1059.59 ( 2350.2 ) | 589.1 ( 196.06 ) | 9.2 ( 7.62 ) | 3 | Moderate |
| Left-to-right cardiac shunt | 4 | 20.54 ( 7.65 - 55.14 ) | 20.53 ( 73.2 ) | 20.24 ( 8.86 ) | 4.34 ( 3.04 ) | 3 | Moderate |
| Upper respiratory tract inflammation | 4 | 6.55 ( 2.45 - 17.49 ) | 6.55 ( 18.71 ) | 6.52 ( 2.87 ) | 2.71 ( 1.41 ) | 2 | Low |
| Glomerular filtration rate abnormal | 4 | 4.77 ( 1.79 - 12.73 ) | 4.77 ( 11.87 ) | 4.76 ( 2.09 ) | 2.25 ( 0.96 ) | 2 | Low |
| Viral load | 4 | 39.54 ( 14.63 - 106.9 ) | 39.54 ( 145.89 ) | 38.42 ( 16.72 ) | 5.26 ( 3.95 ) | 2 | Low |
| Chlamydial infection | 4 | 10.39 ( 3.88 - 27.79 ) | 10.39 ( 33.67 ) | 10.32 ( 4.53 ) | 3.37 ( 2.07 ) | 2 | Low |
| Conversion disorder | 4 | 5.97 ( 2.24 - 15.95 ) | 5.97 ( 16.49 ) | 5.95 ( 2.62 ) | 2.57 ( 1.28 ) | 2 | Low |
| Ectopic kidney | 4 | 80.28 ( 29.26 - 220.27 ) | 80.27 ( 295.24 ) | 75.74 ( 32.55 ) | 6.24 ( 4.91 ) | 2 | Low |
| Antiviral drug level below therapeutic | 4 | 230.37 ( 79.67 - 666.18 ) | 230.35 ( 778.08 ) | 196.37 ( 80.76 ) | 7.62 ( 6.22 ) | 2 | Low |
| Acarodermatitis | 4 | 6.11 ( 2.29 - 16.32 ) | 6.11 ( 17.02 ) | 6.09 ( 2.68 ) | 2.61 ( 1.31 ) | 2 | Low |
| Congenital acrochordon | 4 | 147.18 ( 52.38 - 413.54 ) | 147.16 ( 522.62 ) | 132.55 ( 55.84 ) | 7.05 ( 5.68 ) | 2 | Low |
| Congenital choroid plexus cyst | 4 | 25.11 ( 9.34 - 67.53 ) | 25.11 ( 90.87 ) | 24.66 ( 10.78 ) | 4.62 ( 3.32 ) | 2 | Low |
| Congenital naevus | 4 | 48.61 ( 17.92 - 131.86 ) | 48.6 ( 179.9 ) | 46.92 ( 20.36 ) | 5.55 ( 4.23 ) | 2 | Low |
| Impetigo | 4 | 5.65 ( 2.12 - 15.08 ) | 5.65 ( 15.24 ) | 5.63 ( 2.47 ) | 2.49 ( 1.2 ) | 2 | Low |
| Foetal monitoring abnormal | 4 | 40.45 ( 14.96 - 109.39 ) | 40.44 ( 149.31 ) | 39.27 ( 17.08 ) | 5.3 ( 3.98 ) | 2 | Low |
| Pharyngeal chlamydia infection | 4 | 1766.2 ( 395.27 - 7891.95 ) | 1765.98 ( 3023.97 ) | 757.42 ( 216.44 ) | 9.56 ( 7.92 ) | 2 | Low |
| High density lipoprotein increased | 4 | 8.59 ( 3.21 - 22.96 ) | 8.59 ( 26.64 ) | 8.54 ( 3.75 ) | 3.09 ( 1.8 ) | 2 | Low |
| Food interaction | 4 | 4.8 ( 1.8 - 12.82 ) | 4.8 ( 12 ) | 4.79 ( 2.11 ) | 2.26 ( 0.97 ) | 2 | Low |
| Latent syphilis | 4 | 378.47 ( 124.57 - 1149.87 ) | 378.42 ( 1171.11 ) | 294.55 ( 116.24 ) | 8.2 ( 6.75 ) | 2 | Low |
| Intercepted product dispensing error | 4 | 5.5 ( 2.06 - 14.68 ) | 5.5 ( 14.65 ) | 5.48 ( 2.41 ) | 2.45 ( 1.16 ) | 2 | Low |
| Cd4 lymphocyte percentage decreased | 4 | 151.39 ( 53.8 - 425.98 ) | 151.37 ( 536.22 ) | 135.95 ( 57.2 ) | 7.09 ( 5.72 ) | 2 | Low |
| Blood triglycerides decreased | 4 | 23.44 ( 8.72 - 63.01 ) | 23.44 ( 84.45 ) | 23.05 ( 10.08 ) | 4.53 ( 3.22 ) | 2 | Low |
| Labelled drug-drug interaction issue | 4 | 5.65 ( 2.12 - 15.1 ) | 5.65 ( 15.26 ) | 5.63 ( 2.48 ) | 2.49 ( 1.2 ) | 2 | Low |
| Paranasal cyst | 4 | 31.17 ( 11.56 - 84.01 ) | 31.16 ( 114.1 ) | 30.47 ( 13.29 ) | 4.93 ( 3.62 ) | 2 | Low |
| Thalassaemia beta | 4 | 101.9 ( 36.85 - 281.75 ) | 101.88 ( 371.03 ) | 94.68 ( 40.43 ) | 6.56 ( 5.22 ) | 2 | Low |
| Pancreatic toxicity | 4 | 62.34 ( 22.87 - 169.93 ) | 62.33 ( 230.53 ) | 59.57 ( 25.74 ) | 5.9 ( 4.57 ) | 2 | Low |
| Cryptosporidiosis infection | 4 | 14.05 ( 5.25 - 37.64 ) | 14.05 ( 47.99 ) | 13.92 ( 6.1 ) | 3.8 ( 2.5 ) | 2 | Low |
| Discharge | 4 | 4.68 ( 1.75 - 12.49 ) | 4.68 ( 11.53 ) | 4.67 ( 2.05 ) | 2.22 ( 0.93 ) | 2 | Low |
| Central nervous system immune reconstitution inflammatory response | 3 | 113.54 ( 34.92 - 369.18 ) | 113.53 ( 308.19 ) | 104.64 ( 39.01 ) | 6.71 ( 5.19 ) | 5 | Moderate |
| Death neonatal | 3 | 5.26 ( 1.69 - 16.34 ) | 5.26 ( 10.3 ) | 5.24 ( 2.03 ) | 2.39 ( 0.94 ) | 5 | Moderate |
| Immune reconstitution inflammatory syndrome associated kaposi's sarcoma | 3 | 63.08 ( 19.81 - 200.87 ) | 63.07 ( 174.93 ) | 60.25 ( 22.86 ) | 5.91 ( 4.43 ) | 4 | Moderate |
| Anomalous pulmonary venous connection | 3 | 24.53 ( 7.83 - 76.86 ) | 24.53 ( 66.47 ) | 24.1 ( 9.27 ) | 4.59 ( 3.13 ) | 4 | Moderate |
| Encephalitis cytomegalovirus | 3 | 13.42 ( 4.3 - 41.87 ) | 13.42 ( 34.15 ) | 13.3 ( 5.13 ) | 3.73 ( 2.28 ) | 4 | Moderate |
| Lissencephaly | 3 | 137.03 ( 41.74 - 449.86 ) | 137.02 ( 367.09 ) | 124.26 ( 45.96 ) | 6.96 ( 5.43 ) | 4 | Moderate |
| Foetal heart rate abnormal | 3 | 7.2 ( 2.31 - 22.39 ) | 7.2 ( 15.93 ) | 7.16 ( 2.77 ) | 2.84 ( 1.39 ) | 4 | Moderate |
| Meningitis tuberculous | 3 | 10.03 ( 3.22 - 31.25 ) | 10.03 ( 24.22 ) | 9.97 ( 3.85 ) | 3.32 ( 1.87 ) | 4 | Moderate |
| Central nervous system inflammation | 3 | 18.57 ( 5.94 - 58.04 ) | 18.57 ( 49.18 ) | 18.32 ( 7.06 ) | 4.2 ( 2.74 ) | 4 | Moderate |
| Hypertrophy | 3 | 13.07 ( 4.19 - 40.76 ) | 13.07 ( 33.11 ) | 12.95 ( 5 ) | 3.7 ( 2.24 ) | 4 | Moderate |
| Porphyria non-acute | 3 | 15.77 ( 5.05 - 49.23 ) | 15.77 ( 41.01 ) | 15.59 ( 6.02 ) | 3.96 ( 2.51 ) | 3 | Moderate |
| Thalamus haemorrhage | 3 | 5.85 ( 1.88 - 18.19 ) | 5.85 ( 12.02 ) | 5.83 ( 2.26 ) | 2.54 ( 1.1 ) | 3 | Moderate |
| Pneumonia cryptococcal | 3 | 5.97 ( 1.92 - 18.55 ) | 5.97 ( 12.35 ) | 5.94 ( 2.3 ) | 2.57 ( 1.12 ) | 3 | Moderate |
| Blood hiv rna decreased | 3 | 1986.91 ( 331.98 - 11891.71 ) | 1986.73 ( 2381.67 ) | 795.29 ( 177.96 ) | 9.64 ( 7.77 ) | 3 | Moderate |
| Mycobacterium avium complex immune restoration disease | 3 | 331.15 ( 93.44 - 1173.56 ) | 331.12 ( 789.9 ) | 265.1 ( 91.97 ) | 8.05 ( 6.44 ) | 3 | Moderate |
| Congenital hydronephrosis | 3 | 8.06 ( 2.59 - 25.08 ) | 8.06 ( 18.44 ) | 8.02 ( 3.1 ) | 3 ( 1.55 ) | 3 | Moderate |
| Tuberculoma of central nervous system | 3 | 30.57 ( 9.73 - 96.02 ) | 30.57 ( 83.86 ) | 29.9 ( 11.47 ) | 4.9 ( 3.44 ) | 3 | Moderate |
| Hyperglycaemic hyperosmolar nonketotic syndrome | 3 | 5.47 ( 1.76 - 17.01 ) | 5.47 ( 10.92 ) | 5.45 ( 2.11 ) | 2.45 ( 1 ) | 3 | Moderate |
| Lymph node tuberculosis | 3 | 8.66 ( 2.78 - 26.94 ) | 8.66 ( 20.19 ) | 8.61 ( 3.33 ) | 3.11 ( 1.66 ) | 3 | Moderate |
| Retroviral rebound syndrome | 3 | 248.36 ( 72.36 - 852.42 ) | 248.34 ( 622.35 ) | 209.29 ( 74.58 ) | 7.71 ( 6.13 ) | 3 | Moderate |
| Endocardial fibroelastosis | 3 | 172.77 ( 51.87 - 575.47 ) | 172.76 ( 453.18 ) | 152.94 ( 55.88 ) | 7.26 ( 5.71 ) | 3 | Moderate |
| Developmental hip dysplasia | 3 | 5.8 ( 1.87 - 18.03 ) | 5.8 ( 11.87 ) | 5.78 ( 2.24 ) | 2.53 ( 1.08 ) | 3 | Moderate |
| Placenta praevia | 3 | 8.09 ( 2.6 - 25.18 ) | 8.09 ( 18.54 ) | 8.05 ( 3.11 ) | 3.01 ( 1.56 ) | 3 | Moderate |
| Laryngomalacia | 3 | 16.84 ( 5.39 - 52.59 ) | 16.84 ( 44.13 ) | 16.64 ( 6.42 ) | 4.06 ( 2.6 ) | 3 | Moderate |
| Foetal disorder | 3 | 9.16 ( 2.94 - 28.5 ) | 9.16 ( 21.64 ) | 9.1 ( 3.52 ) | 3.19 ( 1.74 ) | 3 | Moderate |
| Congenital hiv infection | 3 | 331.15 ( 93.44 - 1173.56 ) | 331.12 ( 789.9 ) | 265.1 ( 91.97 ) | 8.05 ( 6.44 ) | 3 | Moderate |
| Carotid arteriosclerosis | 3 | 5.72 ( 1.84 - 17.77 ) | 5.72 ( 11.63 ) | 5.7 ( 2.21 ) | 2.51 ( 1.06 ) | 3 | Moderate |
| Foetal heart rate deceleration abnormality | 3 | 6.65 ( 2.14 - 20.66 ) | 6.64 ( 14.31 ) | 6.62 ( 2.56 ) | 2.73 ( 1.28 ) | 3 | Moderate |
| Vasogenic cerebral oedema | 3 | 7.5 ( 2.41 - 23.32 ) | 7.5 ( 16.8 ) | 7.46 ( 2.89 ) | 2.9 ( 1.45 ) | 3 | Moderate |
| Renal tubular atrophy | 3 | 7.03 ( 2.26 - 21.87 ) | 7.03 ( 15.44 ) | 7 ( 2.71 ) | 2.81 ( 1.36 ) | 3 | Moderate |
| Proctitis chlamydial | 3 | 662.3 ( 165.63 - 2648.36 ) | 662.24 ( 1320.49 ) | 441.83 ( 138.55 ) | 8.79 ( 7.08 ) | 3 | Moderate |
| Holoprosencephaly | 3 | 42.27 ( 13.39 - 133.45 ) | 42.27 ( 117.14 ) | 40.99 ( 15.67 ) | 5.36 ( 3.89 ) | 3 | Moderate |
| Amniotic cavity infection | 3 | 6.71 ( 2.16 - 20.87 ) | 6.71 ( 14.51 ) | 6.68 ( 2.59 ) | 2.74 ( 1.29 ) | 3 | Moderate |
| Low set ears | 3 | 16.91 ( 5.41 - 52.81 ) | 16.91 ( 44.34 ) | 16.71 ( 6.44 ) | 4.06 ( 2.61 ) | 3 | Moderate |
| Hepatitis alcoholic | 3 | 20.81 ( 6.65 - 65.09 ) | 20.8 ( 55.68 ) | 20.5 ( 7.89 ) | 4.36 ( 2.9 ) | 3 | Moderate |
| Hypereosinophilic syndrome | 3 | 10.86 ( 3.49 - 33.82 ) | 10.86 ( 26.63 ) | 10.78 ( 4.16 ) | 3.43 ( 1.98 ) | 3 | Moderate |
| Gastric fistula | 3 | 11.72 ( 3.76 - 36.53 ) | 11.72 ( 29.16 ) | 11.63 ( 4.49 ) | 3.54 ( 2.09 ) | 3 | Moderate |
| Renal aplasia | 3 | 8.89 ( 2.86 - 27.67 ) | 8.89 ( 20.87 ) | 8.84 ( 3.42 ) | 3.14 ( 1.69 ) | 3 | Moderate |
| Necrotic lymphadenopathy | 3 | 49.06 ( 15.5 - 155.32 ) | 49.05 ( 136.18 ) | 47.34 ( 18.05 ) | 5.56 ( 4.09 ) | 3 | Moderate |
| Chronic hepatitis b | 3 | 15.52 ( 4.97 - 48.45 ) | 15.52 ( 40.29 ) | 15.35 ( 5.92 ) | 3.94 ( 2.49 ) | 3 | Moderate |
| Solar dermatitis | 3 | 17.2 ( 5.51 - 53.73 ) | 17.2 ( 45.19 ) | 16.99 ( 6.55 ) | 4.09 ( 2.63 ) | 2 | Low |
| Stab wound | 3 | 15.58 ( 4.99 - 48.64 ) | 15.58 ( 40.46 ) | 15.41 ( 5.95 ) | 3.95 ( 2.49 ) | 2 | Low |
| Cat scratch disease | 3 | 14.29 ( 4.58 - 44.59 ) | 14.29 ( 36.69 ) | 14.15 ( 5.46 ) | 3.82 ( 2.37 ) | 2 | Low |
| Waist circumference increased | 3 | 5.43 ( 1.75 - 16.87 ) | 5.43 ( 10.79 ) | 5.41 ( 2.09 ) | 2.44 ( 0.99 ) | 2 | Low |
| Cranial nerve disorder | 3 | 8.55 ( 2.75 - 26.6 ) | 8.55 ( 19.86 ) | 8.5 ( 3.29 ) | 3.09 ( 1.64 ) | 2 | Low |
| Biopsy liver | 3 | 14.56 ( 4.67 - 45.42 ) | 14.55 ( 37.46 ) | 14.41 ( 5.56 ) | 3.85 ( 2.4 ) | 2 | Low |
| Enzyme abnormality | 3 | 15.83 ( 5.07 - 49.42 ) | 15.83 ( 41.19 ) | 15.66 ( 6.04 ) | 3.97 ( 2.51 ) | 2 | Low |
| Choluria | 3 | 18.74 ( 6 - 58.59 ) | 18.74 ( 49.69 ) | 18.5 ( 7.13 ) | 4.21 ( 2.75 ) | 2 | Low |
| Glomerular filtration rate increased | 3 | 6.5 ( 2.09 - 20.22 ) | 6.5 ( 13.9 ) | 6.48 ( 2.51 ) | 2.7 ( 1.25 ) | 2 | Low |
| Toxoplasmosis | 3 | 5.61 ( 1.81 - 17.45 ) | 5.61 ( 11.32 ) | 5.59 ( 2.17 ) | 2.48 ( 1.04 ) | 2 | Low |
| Threatened labour | 3 | 14.77 ( 4.73 - 46.1 ) | 14.77 ( 38.09 ) | 14.62 ( 5.64 ) | 3.87 ( 2.42 ) | 2 | Low |
| Pregnancy with advanced maternal age | 3 | 128.19 ( 39.19 - 419.32 ) | 128.18 ( 345.15 ) | 116.95 ( 43.39 ) | 6.87 ( 5.35 ) | 2 | Low |
| Eye symptom | 3 | 14.24 ( 4.57 - 44.43 ) | 14.24 ( 36.54 ) | 14.1 ( 5.44 ) | 3.82 ( 2.36 ) | 2 | Low |
| Creatinine renal clearance increased | 3 | 8.33 ( 2.68 - 25.92 ) | 8.33 ( 19.23 ) | 8.28 ( 3.2 ) | 3.05 ( 1.6 ) | 2 | Low |
| Leiomyoma | 3 | 8.55 ( 2.75 - 26.6 ) | 8.55 ( 19.86 ) | 8.5 ( 3.29 ) | 3.09 ( 1.64 ) | 2 | Low |
| Beta 2 microglobulin increased | 3 | 28.59 ( 9.11 - 89.73 ) | 28.59 ( 78.18 ) | 28 ( 10.75 ) | 4.81 ( 3.34 ) | 2 | Low |
| Hydrocele | 3 | 5.64 ( 1.82 - 17.54 ) | 5.64 ( 11.42 ) | 5.62 ( 2.18 ) | 2.49 ( 1.04 ) | 2 | Low |
| Balanoposthitis | 3 | 6.06 ( 1.95 - 18.83 ) | 6.06 ( 12.61 ) | 6.03 ( 2.34 ) | 2.59 ( 1.15 ) | 2 | Low |
| Urethral discharge | 3 | 60.21 ( 18.93 - 191.51 ) | 60.2 ( 167.07 ) | 57.63 ( 21.89 ) | 5.85 ( 4.37 ) | 2 | Low |
| Blood bilirubin unconjugated increased | 3 | 11.01 ( 3.53 - 34.29 ) | 11.01 ( 27.07 ) | 10.92 ( 4.22 ) | 3.45 ( 2 ) | 2 | Low |
| Complication of pregnancy | 3 | 7.4 ( 2.38 - 23.02 ) | 7.4 ( 16.51 ) | 7.36 ( 2.85 ) | 2.88 ( 1.43 ) | 2 | Low |
| Prolonged pregnancy | 3 | 43.19 ( 13.68 - 136.41 ) | 43.19 ( 119.73 ) | 41.86 ( 15.99 ) | 5.39 ( 3.91 ) | 2 | Low |
| Molluscum contagiosum | 3 | 11.55 ( 3.71 - 36 ) | 11.55 ( 28.66 ) | 11.46 ( 4.43 ) | 3.52 ( 2.07 ) | 2 | Low |
| Exposure via body fluid | 3 | 5.63 ( 1.81 - 17.49 ) | 5.63 ( 11.37 ) | 5.61 ( 2.17 ) | 2.49 ( 1.04 ) | 2 | Low |
| Mucosal hypertrophy | 3 | 33.68 ( 10.71 - 105.93 ) | 33.67 ( 92.75 ) | 32.86 ( 12.6 ) | 5.04 ( 3.57 ) | 2 | Low |
| Burning mouth syndrome | 3 | 9.79 ( 3.14 - 30.48 ) | 9.79 ( 23.49 ) | 9.72 ( 3.76 ) | 3.28 ( 1.83 ) | 2 | Low |
| Pharyngitis bacterial | 3 | 19.38 ( 6.2 - 60.61 ) | 19.38 ( 51.55 ) | 19.12 ( 7.37 ) | 4.26 ( 2.8 ) | 2 | Low |
| Supernumerary nipple | 3 | 66.23 ( 20.77 - 211.19 ) | 66.22 ( 183.54 ) | 63.12 ( 23.92 ) | 5.98 ( 4.49 ) | 2 | Low |
| Tinea versicolour | 3 | 15.4 ( 4.93 - 48.07 ) | 15.4 ( 39.93 ) | 15.24 ( 5.88 ) | 3.93 ( 2.48 ) | 2 | Low |
| Lymphocytic infiltration | 3 | 6.95 ( 2.23 - 21.61 ) | 6.95 ( 15.19 ) | 6.92 ( 2.68 ) | 2.79 ( 1.34 ) | 2 | Low |
| Testicular disorder | 3 | 6.33 ( 2.04 - 19.67 ) | 6.33 ( 13.39 ) | 6.3 ( 2.44 ) | 2.66 ( 1.21 ) | 2 | Low |
| Right ventricular hypertrophy | 3 | 6.83 ( 2.2 - 21.23 ) | 6.83 ( 14.84 ) | 6.8 ( 2.63 ) | 2.76 ( 1.32 ) | 2 | Low |
| Urethritis | 3 | 11.59 ( 3.72 - 36.1 ) | 11.58 ( 28.76 ) | 11.49 ( 4.44 ) | 3.52 ( 2.07 ) | 2 | Low |
| Amoebiasis | 3 | 23.94 ( 7.64 - 74.99 ) | 23.94 ( 64.76 ) | 23.53 ( 9.05 ) | 4.56 ( 3.1 ) | 2 | Low |
| Mycoplasma infection | 3 | 9.2 ( 2.95 - 28.63 ) | 9.2 ( 21.77 ) | 9.14 ( 3.53 ) | 3.19 ( 1.74 ) | 2 | Low |
| Haemangioma congenital | 3 | 13.33 ( 4.28 - 41.58 ) | 13.33 ( 33.89 ) | 13.21 ( 5.1 ) | 3.72 ( 2.27 ) | 2 | Low |
| Urethritis chlamydial | 3 | 794.76 ( 189.92 - 3325.81 ) | 794.69 ( 1486.3 ) | 497.06 ( 150.05 ) | 8.96 ( 7.22 ) | 2 | Low |
| Blood triglycerides abnormal | 3 | 7.05 ( 2.27 - 21.91 ) | 7.05 ( 15.48 ) | 7.01 ( 2.71 ) | 2.81 ( 1.36 ) | 2 | Low |
| Sideroblastic anaemia | 3 | 56.77 ( 17.87 - 180.3 ) | 56.76 ( 157.59 ) | 54.47 ( 20.71 ) | 5.77 ( 4.29 ) | 2 | Low |
| Sexual transmission of infection | 3 | 397.38 ( 109.36 - 1444 ) | 397.35 ( 912.34 ) | 305.88 ( 103.92 ) | 8.26 ( 6.62 ) | 2 | Low |
